# Current landscape and future directions of targeted-alpha-therapy for glioblastoma treatment

**DOI:** 10.7150/thno.106081

**Published:** 2025-03-31

**Authors:** Loris Roncali, François Hindré, Edouard Samarut, Franck Lacoeuille, Audrey Rousseau, Jean-Michel Lemée, Emmanuel Garcion, Michel Chérel

**Affiliations:** 1 Centre for Research in Molecular Medicine and Chronic Diseases (CiMUS), University of Santiago de Compostela; E-15782 Santiago de Compostela, Spain.; 2 University of Angers, INSERM, CNRS, CRCI 2 NA; F-49000 Angers, France.; 3 PRIMEX (Experimental Imagery and Radiobiology Platform), University of Angers, SFR 4208; F-49000 Angers, France.; 4 Nantes University, INSERM, CNRS, CRCI 2 NA; F-44000 Nantes, France.; 5 Department of Neurosurgery & Neurotraumatology, University Hospital of Nantes; F-44093 Nantes, France.; 6 Department of Nuclear Medicine, University Hospital of Angers; F-49000 Angers, France.; 7 Department of Pathology, University Hospital of Angers; F-49000 Angers, France.; 8 Department of Neurosurgery, University Hospital of Angers; F-49000 Angers, France.; 9 PACEM (Platform of Cellular and Molecular Analysis), University of Angers, SFR 4208; F-49000 Angers, France.; 10 Institut de Cancérologie de l'Ouest, Department of Nuclear Medicine; F-44160 Saint-Herblain, France.

**Keywords:** glioblastoma, targeted-alpha-therapy, astatine-211, actinium-225, bismuth-213

## Abstract

Glioblastoma (GB) is the most aggressive malignancy of the central nervous system. Despite two decades of intensive research since the establishment of the standard of care, emerging strategies have yet to produce consistent satisfactory outcomes. Because of its specific localisation and intricate characteristics, GB is a uniquely regulated solid tumour with a strong resistance to therapy. Advances in targeted radionuclide therapy (TRT), particularly with the introduction of a-emitting radionuclides, have unveiled potential avenues for the management of GB. Recent preclinical and clinical studies underscored promising advancements for targeted-α-therapy (TAT), but these therapeutic approaches exhibit a vast design heterogeneity, encompassing diverse radionuclides, vectors, target molecules, and administration modalities. This review seeks to critically assess the therapeutic landscape of GB through the perspective of TAT. Here, the focus is made on the advancements and limitations of *in vivo* explorations, pilot studies, and clinical trials, to determine the best directions for future investigations. In doing so, we hope to identify existing challenges and draw insights that might pave the way towards a more effective therapeutic approach.

## Introduction

Glioblastoma (GB) is the most aggressive form of brain tumours. Classified as a grade 4 astrocytoma by the World Health Organisation 1, its annual incidence is 3.27 cases per 100,000 people 2. Since 2005, the standard of care consists in maximal surgical resection followed by the Stupp protocol, which combines radiotherapy and chemotherapy with temozolomide (TMZ) 3. However, this approach is limited by a 15-month median overall survival. In the past two decades, advancements have mainly relied on this protocol and remain insufficient. During this period, bevacizumab, an anti-angiogenic monoclonal antibody approved by the Food and Drug Administration (FDA), has improved the quality of life of patients but has failed to prolong survival 4,5. Additionally, tumour-treating fields (TTFs, Optune^®^), using low-intensity and intermediate-frequency electric fields, constitute a noteworthy addition to GB treatment but still lack curative potential 6,7. Lastly, immunotherapy, despite showing preclinical promise, has yet to achieve substantial success in Phase III clinical trials 8.

The resistance of GB to therapy results from its multifaceted heterogeneity 9,10 which is driven by factors such as genomic instability 11, hypoxic niches 12, and GB stem-like cells (GSLCs) 13, establishing a specific tumour microenvironment (TME) and strengthening immunosuppression 14 and tumour infiltration 15. In addition, only 20 to 30% of patients are deemed suitable for surgery 16. In these cases, the resection cavity impedes the local administration of post-operative treatments, leading to a recurrence rate of 90% 17. Similarly, the blood-brain barrier (BBB) increases treatment challenges by shielding the brain from systemic circulation, effectively blocking the administration of most small-molecule drugs 18.

Targeted radionuclide therapy (TRT) holds promise for breakthroughs in cancer treatment. This strategy involves the administration of high-energy radionuclides either intravenously or locoregionally. Radionuclides can selectively accumulate at tumour sites through passive targeting - using their intrinsic chemical properties or nanoparticles (NPs) carriers - or through active targeting, achieved by their conjugation with vectors such as monoclonal antibodies (mAbs) or peptides that specifically bind to tumour antigens. This accumulation results in both direct and indirect cellular impacts. The direct effects arise from energy transfer, leading to phenomena such as DNA damage and crossfire effects. Indirect effects, on the other hand, stem from the generation of reactive oxygen species (ROS) *via* H_2_O radiolysis and radiation-induced bystander effects. These effects involve signal propagation from radiated to neighbouring cells, triggering apoptosis in cells not immediately exposed to ionising radiation. In parallel to these local communicative effects, the long-distance abscopal effect could potentially elicit immune response at a distant tumour site extending outside the treated volume 19.

Early studies focused on β^-^ radionuclides like iodine-131 (^131^I), yttrium-90 (^90^Y), or lutetium-177 (^177^Lu), due to their shorter tissue penetration (1-10 mm) compared to X-rays used in external beam radiation 20-25. These radionuclides exhibit an emission energy between 0.1 and 2.3 MeV and a linear energy transfer (LET; the amount of energy deposited per length unit) of 0.2 keV/µm. In the early 2000s, successful clinical trials led to the approval of treatments for non-Hodgkin lymphoma based on an anti-CD20 mAb, such as ^90^Y-ibritumomab tiuxetan (Zevalin^®^) 26 and ^131^I-tositumomab (Bexxar^®^) 27. In 2018, the FDA approved the combination of the somatostatin analogue DOTATATE with ^177^Lu (^177^Lu-DOTATATE, Lutathera^®^) for the treatment of gastroenteropancreatic neuroendocrine tumours (GEP-NETs) 28. Additionally, ^177^Lu-labelled PSMA-617 (^177^Lu vipivotide tetraxetan, Pluvicto^®^) was greenlit in 2022 for the treatment of adult patients with prostate-specific membrane antigen (PSMA)-positive metastatic castration-resistant prostate cancer (mCRPC) 29.

However, the substantial penetration of β^-^-emitters does not always guarantee the preservation of healthy tissue around irradiated areas 30. α-emitters, with their shorter tissue penetration (50-100 μm), higher energy emissions (2-10 MeV), higher linear energy transfer (100 keV/μm) 31, and maintained efficacy in hypoxic conditions 32, offer a potential shift in nuclear medicine. Significant clinical outcomes have already been demonstrated, leading to the FDA approval of the radium-223 dichloride (^223^Ra; Xofigo^®^) in 2013 for the treatment of mCRPC 33. For GB, α-emitters could effectively eliminate both primary tumours and residual cells post-surgery while sparing healthy tissues. Recent pilot studies and clinical trials confirmed the safety and efficacy of targeted-α-therapies (TATs) using astatine-211 (^211^At), bismuth-213 (^213^Bi), and actinium-225 (^225^Ac) in GB scenarios, with subsequent research showing improved survival rates in GB rodent models.

This review explores the rise and potential of TAT in the treatment of GB, providing a comprehensive analysis of both preclinical *in vivo* studies and clinical investigations. Specifically, it examines key preclinical aspects in the design of published studies, including radionuclide selection, vectorisation strategies, administration methods, and dose selection. By drawing conclusions from the existing literature, we aim to encourage the standardisation of investigation methods for TAT in GB. The review integrates nuclear medicine with target biology, anti-tumour responses, and mechanisms of intrinsic and extrinsic resistance. Given the intricate challenge of GB as a whole-brain tumour, we aim to guide future TAT research through thoughtful experimental design and to highlight potential avenues for therapeutic innovation.

## Primary α-emitters for targeted-α-therapy in glioblastoma

**Figure 1:**
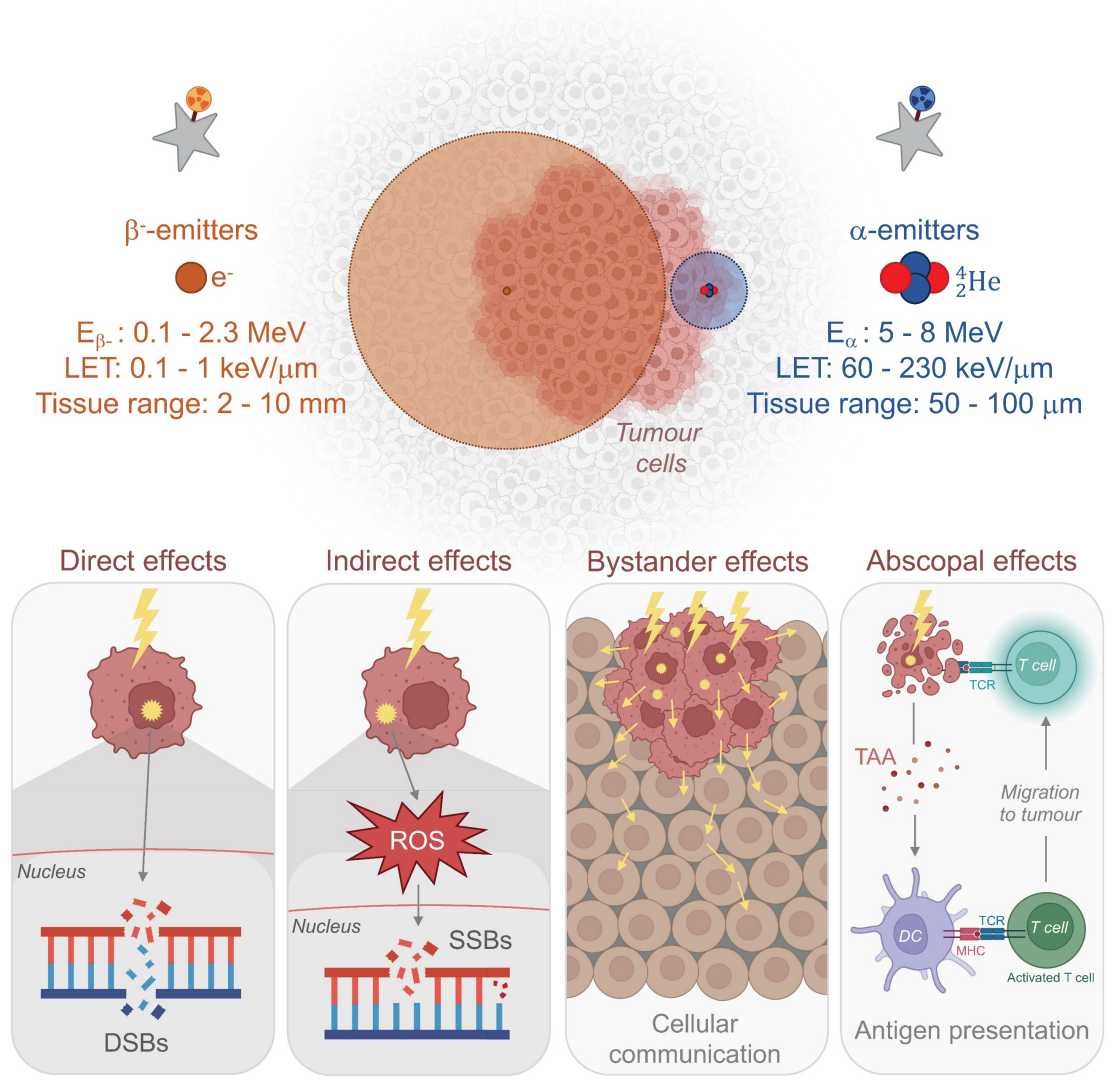
** Properties and radiobiological effects of β^-^ and α-emitters.** DSB: double-strand break; E: energy; LET: linear energy transfer; MHC: major histocompatibility complex; ROS: reactive oxygen species; SSB: single-strand break; TAA: tumour-associated antigen; TCR: T cell receptor. (Created with Biorender - biorender.com).

Considering the inherent physical, cellular, molecular, and microenvironmental challenges associated with GB, the task for α-emitters to surpass β^-^-emitters and achieve a curative effect with reduced toxicity is substantial (Fig. 1). In this section, we introduce the α-emitting radionuclides used in preclinical *in vivo* studies and clinical trials for the treatment of GB. Their availability, production and chemistry have already been extensively reviewed 34-36 and will be briefly discussed here. All *in vivo* preclinical studies of TAT for GB are summarised in Tables 1 and 2 with a focus made on the methodology of each study. Clinical trials and pilot studies are presented in the Table 3.

### Astatine-211

**Figure 2:**
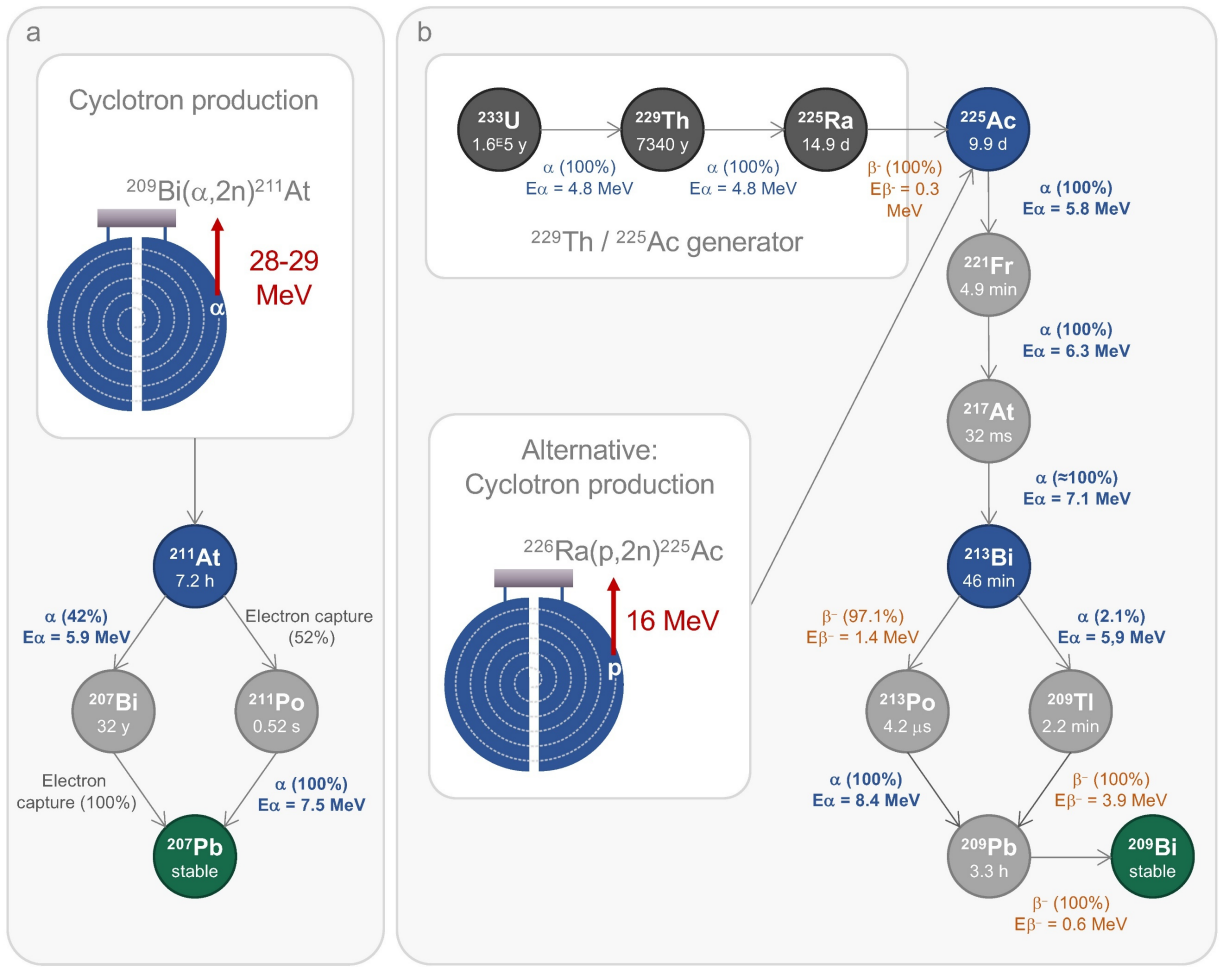
** Main production routes and decay schemes of ^211^At (a), ^225^Ac, and ^213^Bi (b).** Ac: actinium; At: astatine; Bi: bismuth, E: energy; Fr: francium; Pb: lead; p: proton; Po: polonium; Ra: radium; Th: thorium; Tl: thallium; U: uranium.

^211^At undergoes a branching decay process, with each pathway resulting in the release of an α particle, making it a 100% α-emitter. In this decay scheme, ^211^At directly accounts for 42% of the α emission, releasing an energy of 5.9 MeV and subsequently decaying into ^207^Bi. The remaining α particles (7.5 MeV) are emitted by its second daughter radionuclide, polonium-211 (^211^Po). The entire decay sequence of ^211^At results in the stable isotope lead-207 (^207^Pb) (Fig. 2a). This single α emission reduces potential issues related to the uncontrolled spread, as daughter radionuclides may dissociate from their vector during decay. Moreover, the daughter radionuclides produced from ^211^At decay exhibit significantly reduced radiotoxicity. Thus, ^211^Po has an extremely short half-life of 0.52 s, and the generation of ^207^Bi is negligible. Furthermore, with its limited tissue penetration range of 50-90 μm and a half-life of 7.21 h, ^211^At is ideally suited for patient application, as residual radioactivity drops to less than 1% after 48 h. Nonetheless, the half-life of ^211^At is long enough to accommodate multi-step radiolabelling procedures. It is also important to note that ^211^At emits X-rays in the range of 72-92 keV, enabling detection through single-photon emission computed tomography (SPECT) 35.

The standard method to produce ^211^At relies on the nuclear reaction ^209^Bi(α,2n)²¹¹At, which occurs when a natural bismuth target is bombarded with an α particle beam. This reaction requires a cyclotron capable of delivering over 20 MeV of incident energy. This process also generates ^210^At, in a quantity depending on the incident energy level, which poses a significant toxicity risk due to its decay into ^210^Po. As an α-emitter with a half-life of 138 days, ^210^Po is known for its high toxicity, even at low doses 37. Therefore, the optimal incident energy ranges between 28 and 29 MeV to minimise the production of ^210^At while achieving a satisfactory yield of ^211^At 38. Currently, 15 cyclotrons across the USA, Europe, and Asia meet the necessary criteria to carry out this reaction. Projections suggest that up to 30 cyclotrons will be operational in the coming years 36,39,40. Although the current supply does not yet meet the steadily rising demand driven by upcoming clinical needs, government initiatives have been addressing this gap for several years. The recently formed World Astatine Community, emerging from European, American, and Japanese networks, aims to unify global efforts by facilitating the sharing of ^211^At production technology. Furthermore, advances in radiolabelling chemistry and newly proposed methods now allow for the development of novel ^211^At-based molecules for radionuclide therapies. These advancements in radiochemistry have been extensively reviewed recently by Vanermen *et al.* 41.

*Preclinical studies.* Discovered in 1940 42, ^211^At gained renewed interest in the 1980s for developing targeted cancer therapies. After promising clinical results with ^131^I radiopharmaceuticals in GB 20,21,24, research shifted towards ^211^At to develop less toxic treatments, primarily by radiolabelling mAbs targeting tenascin-C (81C6 mAb) and epidermal growth factor receptor variant III (EGFRvIII; L8A4 mAb). Tenascin-C, a component of the extracellular matrix, is overexpressed in 90% of GB cases and is associated with poor prognosis 43,44. Concurrently, the active mutant EGFRvIII was identified as a key target due to its overexpression in GB and its role in angiogenesis, cellular migration, and proliferation 45,46. A substantial challenge in this research was to address the deastatination of *in vitro* synthesised conjugates. In 1989, Zalutsky *et al.* developed a technique that not only reduced deastatination but also preserved the *in vitro* immunoreactivity of labelled mAbs or F(ab')_2_ fragments 47. Preclinical data in mice enabled the determination of the lethal dose for 10% of animals (LD_10_) to be 46 kBq/g for female mice and 102 kBq/g for males, 360 days after injection of ^211^At-labelled 81C6 mAb (^211^At-81C6) 48-53. These studies highlighted the primary toxicity risks related to the potential deastatination of the mAb *in vivo*, with the [^211^At]astatide accumulating in the stomach, spleen, thyroid, and lungs, in that order 54. These findings led to the first clinical trial in 2008, aimed at assessing safety and feasibility of intracranial injection of ^211^At-81C6 in patients with GB (as detailed in the next section) 55.

Since then, successful astatination of new vectors has been achieved to test several targets in GB, including the L-type amino acid transporter 1 receptor (LAT1R) 56,57, poly(ADP-ribose) polymerase (PARP) 58, fibroblast activation protein (FAP) 59-61, vascular endothelial growth factor (VEGF), integrins 62, and more recently syndecan-1 (SDC1) 63. Additionally, ^211^At encapsulation in gold NPs (AuNPs) has been explored with the potential for further functionalisation with targeting vectors such as mAbs or peptides 64,65. Overall, the various preclinical approaches developed over the past decade have demonstrated satisfactory stability of ^211^At conjugates both *in vitro* and *in vivo*. Of the 14 studies conducted with ^211^At on GB rodent models, 11 utilised subcutaneous murine GB models while only three used orthotopic models, with most investigations administering TAT *via* the tail vein. Syngeneic models remain underrepresented, with only two publications to date involving both orthotopic and syngeneic models: the studies by Borrmann *et al.* in 2013 and Roncali *et al.* in 2024 (Table 1) 56,63.

Retention of radioactivity within the tumour is critical for ensuring therapeutic efficacy, yet this aspect was inconsistently addressed across studies. Given the use of both ectopic and orthotopic tumour models and different routes of administration (intravenous, intratumoural, intraperitoneal), the comparison of biodistribution data is limited. For intravenous administration, maximum values ranged from approximately 0.4 %ID/g (90 min after injection) to 6.4 %ID/g (after 6 h) in the tumour 56,60. As expected, intratumoural administration demonstrated superior retention. Notably, Ma *et al.* reported the highest intratumoural retention to date in a subcutaneous GB model, achieving over 130 %ID/g within 30 min following intratumoural injection of ^211^At-labelled FAP inhibitor (FAPi), though this dropped below 20% after just 2 h 59. Our team also replicated the biodistribution of ^211^At in an orthotopic GB model using ^125^I, achieving comparable brain retention of more than 150 %ID/g 2 h post-intratumoural injection, with enhanced retention when coupled with the anti-SDC1 mAb 9E7.4 63.

All strategies have demonstrated the ability to inhibit tumour growth, and some of them showed significantly enhanced survival while maintaining low systemic toxicity 56,59,60,62,63. To date, only the locoregional approach using ^211^At coupled with the anti-SDC1 mAb 9E7.4 (^211^At-9E7.4) has achieved long-term remission in treated animals, with a protection of long-term survivors against tumour rechallenge 63. Currently, the impact of ^211^At on the GB microenvironment remains poorly understood. In the study of Roncali *et al.*, detection of a memory immune response suggested initial immune activation, even though it could not be fully characterised 63. Similarly, Dabagian *et al.* observed increased macrophage phagocytic activity and CD4^+^ T cell activation in a preliminary immunohistochemical study in mouse brains treated with ^211^At-MM4 58. These findings highlight the need for further research, particularly in syngeneic orthotopic models, to validate these responses and investigate the full therapeutic potential of ^211^At-based TAT and its effects on the GB microenvironment.

*Clinical studies*. To date, the only clinical trial using ^211^At for the treatment of GB was conducted by Zalutsky *et al*. and completed in 2005 (NCT00003461). This study involved 18 patients with recurrent brain tumours, all of whom had previously undergone resection surgery and external beam radiation therapy, with 44% having also received prior chemotherapy. After surgical resection, the patients received a single dose of 71-347 MBq of ^211^At-81C6 *via* a Rickham reservoir connected to a catheter placed in the resection cavity. Following TAT injection, 14 patients (78%) received chemotherapy. Initially, 96.7 ± 3.6% of ^211^At decays occurred within the resection cavity, with an estimated total residence time of 10.05 ± 0.4 h. The procedure was well-tolerated, with no grade 3 or higher neurotoxicity observed. Six patients experienced grade 2 neurotoxicity, which resolved within six weeks in five cases. The maximum tolerated dose (MTD) was not reached in this study. Encouraging median survival rates were reported: 13.5 months for all patients, 13 months for the 14 patients with GB, and 29 months for those with astrocytoma or oligodendroglioma 55. These median survival times were comparable to those from a previous clinical study with ^131^I-81C6 20, indicating equivalent efficacy of ^211^At (Table 3).

Currently, there are no ongoing clinical trial using ^211^At for the treatment of GB. However, another clinical trial using ^211^At-labelled antibody for ovarian cancer has been completed (NCT04461457) 66-68, and several trials are ongoing or planned to investigate safety, feasibility and dose escalation in multiple myeloma (NCT04579523, not yet recruiting), acute leukaemia (NCT03670966, suspended; NCT03128034, suspended), thyroid cancer (NCT05275946, recruiting), and prostate cancer (NCT06441994, recruiting). Thus, in the upcoming years, it will be crucial to initiate comprehensive, randomised clinical trials in patients with GB with broader cohorts.

### Actinium-225

The nuclear properties of ^225^Ac present promising potential to develop effective TAT strategies for GB. ^225^Ac emits four α particles during its decay, along with two β^-^ particles. The first three α particles have radiation energies of 5.8, 6.3, and 7.1 MeV as ^225^Ac successively decays into francium-221 (^221^Fr), ^217^At and ^213^Bi. At this point, the decay process branches. In the first branch, 97.1 % of ^213^Bi decays into ^213^Po by emitting a β^-^ particle (1.4 MeV), and ^213^Po then decays into ^209^Pb by emitting an α particle (8.4 MeV). In the second branch (2.1%), ^213^Bi emits an α particle (5.9 MeV) to decay into thallium-209 (^209^Tl), which then emits a β^-^ particle (3.9 MeV) to decay into ^209^Pb. The final β^-^ particle is emitted by ^209^Pb as it decays into to stable ^209^Bi (Fig. 2b) 35. These six emissions provide a powerful tool to design efficient TAT strategies. However, they also introduce challenges, particularly concerning dose control. During decay, secondary radioactivity biodistribution of the daughter radionuclides can occur, potentially causing toxicity in non-targeted tissues 69. Regarding this phenomenon, the main concern is the accumulation of ^213^Bi in the kidneys, which may be dose-limiting for ^225^Ac-based TAT 70,71. A second concern is insufficient chelation of ^225^Ac, resulting in its free diffusion and preferential accumulation in the liver and bones 72. Therefore, chelating ^225^Ac is crucial to mitigate these risks and ensure precise targeting and dose deposition to the tumour. ^225^Ac has a half-life of 9.9 days, making it compatible with macromolecular vectors with extended *in vivo* lifespans. This half-life also simplifies logistical handling and distribution to clinical site but could be too long depending on the pathology being treated. ^225^Ac is also suitable for quantitative imaging, primarily due to its γ emissions at 218 keV from ^221^Fr and 440 keV from ^213^Bi, as well as its emission of bremsstrahlung X-rays and scattered photons 73-75. Recently, quantitative SPECT/CT imaging of ^225^Ac was conducted using a Jaszczak phantom and a 3D-printed model of GB, demonstrating the feasibility of this approach 76.

Current ^225^Ac production is insufficient to meet clinical demand and primarily relies on ^229^thorium (^229^Th)/^225^Ac generators, with ^229^Th originating from the decay of uranium-233 (^233^U). To date, all ^225^Ac used in clinical studies has been produced using this method. Alternative methods for direct production include cyclotron-mediated bombardment of ^226^Ra, or irradiation of ^232^Th with high energy protons, but these methods face challenges related to the availability and handling of ^226^Ra, as well as the production of long half-life ^227^Ac during ^232^Th decay, which require extensive logistical support 77-79. ^227^Ac contamination is a significant issue during the production of ^225^Ac from ^232^Th, particularly because of the challenges associated with isolating ^225^Ac in a pure form. Advances in purification techniques, target design optimisation, and reactor design could mitigate these issues, improving the efficiency and safety of ^225^Ac production. Currently, 14 centres are recognised for their capacity to produce and supply ^225^Ac. Thus, ^225^Ac presents a current annual production capacity of approximately 66 GBq, far below the projected demand of 200-400 GBq per approved therapy. Meanwhile, global investments are underway to scale up ^225^Ac production to meet the rapidly growing clinical demand anticipated over the next decade 36.

*Preclinical studies.* The use of ^225^Ac in cancer treatment has attracted significant attention, particularly through its straightforward complexation with 1-,4-,7-,10-tetraazcy-clododecane-1,4-,7-,10-tetra acetic acid (DOTA), which has become the current gold standard for ^225^Ac chelation 80,81. DOTA is also a key component of the FDA-approved agents [^177^Lu]Lu-DOTATATE and [^68^Ga]Ga-DOTATOC for the treatment and diagnosis of neuroendocrine tumours 28,82. As a result, advancements have been made in treating various cancers, including prostate cancer, neuroendocrine tumours, and acute myeloid leukaemia 83-85. However, progress in the treatment of GB is more recent and still limited. Among the seven related studies discussed here, four employed an orthotopic GB model to evaluate biodistribution, toxicity or therapeutic efficacy (Table 2).

The targeting of proangiogenic integrin α_v_β_3_, which is overexpressed in high-grade gliomas 86-88, was investigated by Pandya *et al.* using the RGDyK peptide vector in a subcutaneous murine GB model. The study focused on the biodistribution and longitudinal imaging of ^225^Ac using Cerenkov luminescence emitted by various ^225^Ac daughter radionuclides 89. Cerenkov luminescence imaging detects ultraviolet light emitted when charged particles exceed the phase velocity of light in a medium 90. This method enabled effective detection of the radionuclide but failed to predict certain toxic effects observed in the animals, particularly nephrotoxicity at high doses. Nonetheless, the treatment was generally well-tolerated with a MTD of 0.04 MBq. Significant tumour regression was observed at doses of 0.01, 0.02, and 0.04 MBq after 14 days 89.

In 2017, Sattiraju *et al.* also targeted α_v_β_3_ in an orthotopic GB model through intratumoural injection. They found that α_v_β_3_-targeted liposomes not only induced permeabilisation of the BBB locally, but also in distant areas unaffected by DNA double-strand breaks (DSB). This suggested a modification of the BBB independent from ^225^Ac radiation 91.

Monomeric vascular endothelial cadherin (VE-cadherin or cadherin 5) is expressed on tumour neovasculature and progenitor endothelial cells and represents another promising target for altering the vascular microenvironment of GB 92,93. Two *in vivo* studies explored its targeting by using the ^225^Ac-labelled E4G10 mAb administered intravenously in a subcutaneous GB model 94,95. Significant BBB modification was observed, including a reduction in the number of epithelial and perivascular cells at activity of 0.074 MBq. This treatment also reduced tumour-related oedema and necrosis zones, and depleted regulatory T cells, which play an immunosuppressive role in GB 95. This approach significantly improved the survival in treated animals, both as a standalone treatment and in combination with TMZ 95.

Targeting interleukine-13 receptor subunit α2 (IL13R_α2_), a well-known GB biomarker involved in tumour progression 96, was also explored using ^225^Ac. In an orthotopic GB model, locoregional convection-enhanced delivery (CED) of a peptide targeting IL13R_α2_ (Pep-1L) labelled with ^225^Ac demonstrated significant progress. An activity of 0.037 MBq resulted in optimal brain retention, substantial tumour growth reduction, and significantly improved survival, with a median survival of 41 days compared to 23 days for mice injected with saline solution 97.

Additionally, AuNPs loaded with ^225^Ac have shown potential as platforms for associating with targeting antibodies or peptides. In an orthotopic murine GB model, these NPs exhibited strong local uptake after intratumoural injection and slow clearance over 12 days. The efficacy was notable since these NPs were not associated with any targeting vector. A previous study with AuNPs labelled with ^177^Lu has already demonstrated strong tumour uptake, with or without targeting 98. However, moderate uptake in the liver, kidneys, and spleen over the same period raises concerns about toxicity, potentially due to clearance through the hepatobiliary pathway or partial release of ^225^Ac from the chelator. A delay in tumour growth over 22 days was achieved after three treatment injections, with a total activity of 15 kBq, emphasising the benefits of dose fractionation in minimising adverse effects 99.

More recently, Wichmann *et al.* investigated the intravenous administration of ^225^Ac-ch806, an anti-EGFR monoclonal antibody, in mice bearing xenografts of U87MG.de2-7 cells, which overexpress the EGFR.de2-7 mutant. The ^225^Ac-ch806 accumulation peaked on day 2 post-injection, reaching 85.4 ± 12.7 %ID/g, with low-level accumulation in the spleen and liver. Mice were treated with a single dose (18.5 kBq, 0.5 μg), which effectively inhibited tumour growth in the U87MG.de2-7 model with a durable tumour-suppressive effect, along with a significantly higher level of DSBs. Treated mice achieved 100% survival at the endpoint of this study 100.

* Clinical studies.* ^225^Ac has been evaluated in only one pilot study for the treatment of GB. Following two pilot studies focused on the use of ^213^Bi, which will be discussed later, Krolicki *et al.* explored the clinical potential of ^225^Ac. They hypothesised enhanced efficacy due to the four α particles emitted by this radionuclide, combined with its considerably longer half-life compared to ^13^Bi (46 min). The study introduced a TAT using ^225^Ac-labelled substance P (^225^Ac-DOTA-SP) to target neurokinin type 1 receptors (NK_1_R) 101, which are overexpressed in GB and play a role in proliferation, invasion, and survival 102-104. The study included 21 patients with recurrent primary (15) and secondary GB (6). All patients had previously undergone the standard therapeutic protocol, including surgery and the Stupp regimen. The dose for this study was informed by earlier human trials, where treatments with three doses of 8 MBq of ^225^Ac-PSMA-617 in patients with prostate cancer was found to be safe and effective. Similarly, three cycles of 18.5 MBq of ^225^Ac-DOTATOC were effective in patients with neuroendocrine tumours 105. In this study, TAT was administered directly into the resection cavity using one or two catheters. Patients received activities ranging from 10 to 30 MBq over one to six cycles, with total activity varying between 10 and 120 MBq. This dose-escalation approach was generally well tolerated. Some patients experienced temporary side effects, including oedema, seizures, aphasia, and hemiparesis. Importantly, no adverse effects related to kidney, liver, or blood were linked to the TAT. While the primary tumour often showed signs of stabilisation, prognosis occasionally worsened due to the emergence of satellite tumours not initially detected by magnetic resonance imaging (MRI). Interestingly, patients with secondary GB had a notably longer survival post-diagnosis compared to those with recurrent primary GB, though both groups exhibited similar progression-free survival and overall survival durations. Although positive survival outcomes were observed, the therapeutic benefit did not directly correlate with the dose administered, and median survival times were less significant than those achieved in earlier studies using ^213^Bi (Table 3).

### Bismuth-213

As previously mentioned, ^213^Bi originates from the decay chain of ^225^Ac and emits one α particle along with two β^-^ particles (Fig. 2b). Its γ emission of 435 keV provides some valuable means for longitudinal tracking using SPECT 35. The short half-life of 45.6 min implies the use of biological vectors with compatible *in vivo* half-lives and poses challenge due to the extended reaction times inherent to radiolabelling chemistry. Fortunately, the existence of a stable isotope of bismuth has facilitated the rapid development of suitable chelators. The CHX-A′′-DTPA ligand has emerged as an optimal choice for ^213^Bi chelation, while DOTA has also proven effective. Both of them form highly stable complexes with strong kinetic stability, thereby reducing the accumulation of free ^213^Bi in kidneys 106,107.

The production of ^213^Bi relies on its parent radionuclide, ^225^Ac, which is loaded into a radionuclide generator. This setup enables the production of ^213^Bi with high specific activity and purity. Importantly, the ^225^Ac required for this process does not need to be pure, as the co-production of ^227^Ac poses no significant issues. The ^225^Ac / ^213^Bi generators typically employ cation and anion exchange or extraction chromatography techniques 108. In clinical studies, the primary production method uses AG MP-50 organic resin, designed for cation exchange, to which ^225^Ac is applied. This system allows to produce radionuclides for several weeks, yielding up to six therapeutic doses per day 109.

*Preclinical Studies*. To date, no *in vivo* investigations involving ^213^Bi have been conducted specifically for GB. However, *in vitro* studies have been conducted on the LN18 GB cell line to assess the effects of ^213^Bi-labelled anti-EGFR mAb on cellular metabolism. In this context, the conversion of hyperpolarised [1-^13^C]pyruvate to [1-^13^C]lactate was monitored using magnetic resonance spectroscopy. The results demonstrated a significant increase in lactate/pyruvate ratio, indicating increased metabolic activity, along with cell death due to the induction of DNA DSBs 110,111. Additionally, several preclinical studies have confirmed the *in vivo* stability of ^213^Bi conjugates. Biodistribution and efficacy studies have also been conducted, primarily using mAb vectors in models such as melanoma 112, multiple myeloma 113, ovarian cancer 114, metastatic breast cancer 115, and bladder carcinoma 116,117.

*Clinical studies.* The first pilot study involving ^213^Bi for the treatment of GB was conducted by Kneifel *et al.* on a very limited cohort 118. In this study, ^213^Bi-DOTAGA-SP was introduced as an alternative to ^90^Y-DOTAGA-SP for some patients to avoid the crossfire effect on critically located brain tumours. Two patients participated: one with GB and another one with grade 2 oligodendroglioma. The patient with GB had previously undergone the standard treatment protocol (surgery, radiotherapy, and chemotherapy) and received an activity of 375 MBq. The patient with oligodendroglioma was administered an activity of 825 MBq after two surgical interventions. Both patients tolerated the treatment well. However, assessing the progression of GB was challenging due to the presence of a residual tumour. In the case of the patient with oligodendroglioma, the resection of a tumour lesion after 33 months revealed radionecrosis with no viable tumour cells, and an additional 34 months passed without recurrence 118.

Another small cohort trial was conducted by Cordier *et al.* to evaluate the efficacy of ^213^Bi as a primary therapeutic modality for patients with critically located GB. In this study, intratumoural placement of one or more catheters was performed, depending on the size and configuration of the tumour, to administer the treatment. The trial involved two patients with GB and three with grade III astrocytoma, who received activities ranging from 1.07 to 29.44 GBq over one to four cycles. Patients showing tumour progression or recurrence subsequently received chemotherapy and/or radiotherapy. The study confirmed the safety and feasibility of the treatment, with MRI showing that TAT induced radionecrosis and allowed for clear delineation of the tumour. Additionally, high retention of ^213^Bi at the injection site was monitored by SPECT 119.

Krolicki *et al.* conducted two similar studies to assess the administration of ^213^Bi-DOTA-SP. In both studies, patients had a catheter placed either in the resection cavity or within the tumour. In the first study, nine patients received treatment consisting in one to six cycles over two months, with a total injected activity ranging from 1.4 to 9.7 GBq. The treatment was generally well-tolerated, with most symptoms being mild and transient, including headaches primarily due to temporary perifocal oedema. Fluid-attenuated inversion recovery (FLAIR) MRI revealed the presence of either oedema or a non-enhancing tumour. The study reported a median progression-free survival of 5.8 months, a median overall survival of 16.4 months from the time of treatment injection, and a median overall survival of 52.3 months from diagnosis 120.

In the second study conducted by Krolicki *et al.*, 20 patients underwent one to seven cycles of ^213^Bi-DOTA-SP treatment. TAT was generally well-tolerated, with minimal side effects. Two patients experienced facial flushing, and one patient had ventricular enhancement. Ten patients had epileptic seizures following the injection, although all had a history of prior seizures. One experienced a brief increase in paresis. Importantly, no severe side effects were observed. The median overall survival was 23.6 months from diagnosis, while the median survival time after recurrence was 10.9 months, with a median progression-free survival of 2.7 months. These results suggest that localised treatment with high activities of ^213^Bi-DOTA-SP offers a promising approach for recurrent GB, providing survival outcomes that compare favourably with conventional treatments. For context, the median survival after the Stupp regimen ranges from 9.7 to 15.9 months 121 (Table 3).

### Promising α-emitters for the treatment of GB

Significant progress has been made in TAT for GB using radionuclides such as ^211^At, ^225^Ac, and ^213^Bi. However, much of the field remains underexplored, with several other α-emitters yet to undergo comprehensive preclinical evaluation for GB therapy. Each radionuclide presents distinct advantages and limitations, with those that are more readily available and easier to handle typically receiving greater research focus. For example, the presence of a stable isotope, as with ^213^Bi, greatly simplifies the radiochemical processes required to produce new stable vectors. Nevertheless, ongoing developments in the global supply of α-emitters and advancements in radiochemistry offer hope for a shift towards exploring these less studied radionuclides. Here, we propose several potential directions for future research involving emerging α-emitters in GB therapy.

^223^Ra represents a promising candidate for TAT with a half-life of 11.43 days and emitting four α and two β^-^ particles. A ^223^Ra-based therapy was evaluated *in vitro* in a GB cell line using a nanozeolite-SP conjugate. This study showed robust retention of the therapeutic agent and significant affinity for NK_1_R, resulting in notable cytotoxicity in the T98G human GB cell line 122. Similarly, ^224^Ra, with a half-life of 3.66 days and an emission profile similar to ^223^Ra, has been explored as an implantable source using ^224^Ra-loaded seeds. This approach was tested in a subcutaneous GB xenograft model, combined with TMZ or bevacizumab. The ^224^Ra treatment significantly slowed tumour growth, with the most effective strategy involving the administration of bevacizumab prior to TAT 123 (Table 2).

^212^Bi (half-life: 60.6 min) is an α-emitter that often receives less attention in therapeutic applications compared to its parent radionuclide, ^212^Pb, due to its relatively short half-life. While ^212^Pb is commonly associated with α emission in TAT, it is, in fact, a 100% β^-^-emitter. The ^212^Pb/^212^Bi pair is frequently used as an *in vivo* radionuclide generator, leveraging the longer half-life of ^212^Pb (10.6 h) to provide a sustained release of ^212^Bi, within a therapeutically optimal time frame. Therefore, ^212^Pb undergoes decay emitting one α particle and two β^-^ particles along its decay chain. Its combination with DOTAMTATE, targeting somatostatin receptors (SSTR) has shown promising results in a mouse model of neuroendocrine tumour 124. Additionally, ^212^Pb has demonstrated efficacy in small cell lung cancer by targeting delta-like ligand 3 (DLL3) 125, and in peritoneal mesothelioma through CD146 targeting 126. It is also under investigation in clinical trials for neuroendocrine tumours (NCT03466216, completed; NCT05153772, active, not yet recruiting) 127. Although the expression of SSTR in GB remains controversial 128-130, it could still be of interest in certain cases, and the stability of this conjugate may facilitate its adaptation for GB therapy. Thus, a clinical trial investigating ^177^Lu-DOTATE in newly diagnosed and recurrent GBs is currently underway (NCT05109728, recruiting) and may lead to the development of analogous approaches employing α emitters.

Other prospective α-emitters, such as ^227^Th (half-life 18.7 days), and terbium-149 (^149^Tb; half-life 4.1 h) present potential for future research in GB treatment but still face production challenges. ^227^Th can be readily chelated and is already being studied for various types of cancers, such as haematologic, breast, colorectal, or prostate cancer 131, with an ongoing clinical trial for mCRPC (NCT03724747, active, not recruiting). Additionally, ^149^Tb is being further developed for improved production and radiochemical purification, showing promising data for future applications 132.

## Preclinical challenges and prospects

Before exploring prospects of TAT, including new research directions and therapeutic combinations, it is crucial to highlight the importance of using appropriate preclinical GB models to achieve meaningful results and facilitate transition to clinical trials. The search for the optimal therapeutic combination - encompassing radionuclide, vector, and target - must continue and will undeniably benefit from the development of new GB models that more accurately reflect clinical realities.

### *In vivo* models

*GB cell lines.* The GB models used for TAT research primarily rely on human GB cell lines for xenograft models, such as U87MG, U251, DF-1, D54MG, as well as rodent cell lines for syngeneic models, including GL261 in mice and C6 and BT4Ca in rats. These cell lines are commonly cultured *in vitro* using two-dimensional culture systems, non-physiological culture media, and enzymatic dissociation. Such practises increase the risk of clonal selection and genetic drift during cultivation, potentially affecting drug response outcomes 133,134. Moreover, prolonged *in vitro* culture exacerbates this issue, leading to a gradual deterioration of the GB signature and the accumulation of genomic duplications and depletions over time. Another important consideration would be to account for key physiological parameters such as normoxia, which ranges from 0.1% to 10% O_2_ in GB, significantly lower than the 21% oxygen typically used in conventional cell culture 135. It is strongly recommended to work under conditions of cerebral normoxia and to carefully select the method of cell dissociation *in vitro*, as trypsin can remove certain surface expressions 136-138. This consideration is particularly critical in the development of targeted therapies. Each GB cell model has its own advantages and limitations 139, and selecting a suitable cell line depends on the specific aspects being studied (*e.g.*, survival, immune responses) and the type of animal model employed.

*Xenograft models.* For xenograft models, U87MG and U251 cell lines are among the most frequently used in research. While U87MG cells are genetically similar to human GB 140, they exhibit limited intratumoural heterogeneity and have a moderate invasive profile 141. Similarly, U251 cells show limited heterogeneity and respond well to chemotherapy and external beam radiotherapy, unlike human GB 142. Given that classical GB cell lines do not fully replicate the complexity of GB, a more accurate approach involves using patient-derived xenografts (PDX), which better reflect the heterogeneity and histology of human GB 143,144. PDX models are created by generating a single cell suspension directly from the tumour sample of a patient and injecting it into a mouse. Additionally, culturing in serum-free media supplemented with fibroblast growth factor b (bFGF) and EGF is a viable option, as it helps maintain the genomic stability of GB cells 145. However, several challenges persist. Accessing patient samples and establishing these models in culture can be difficult and time-consuming. There is also pronounced variability between patients, leading to significant variability between models, which complicates the production of reproducible data. Furthermore, studying immune responses in PDX models requires the use of humanised mice, adding another layer of complexity 146,147. The recent development of fully humanised mice (THX mice), possessing a fully developed and functional human immune system, offers an opportunity to study the immunogenicity of TAT through xenografts of GB 148.

**Figure 3:**
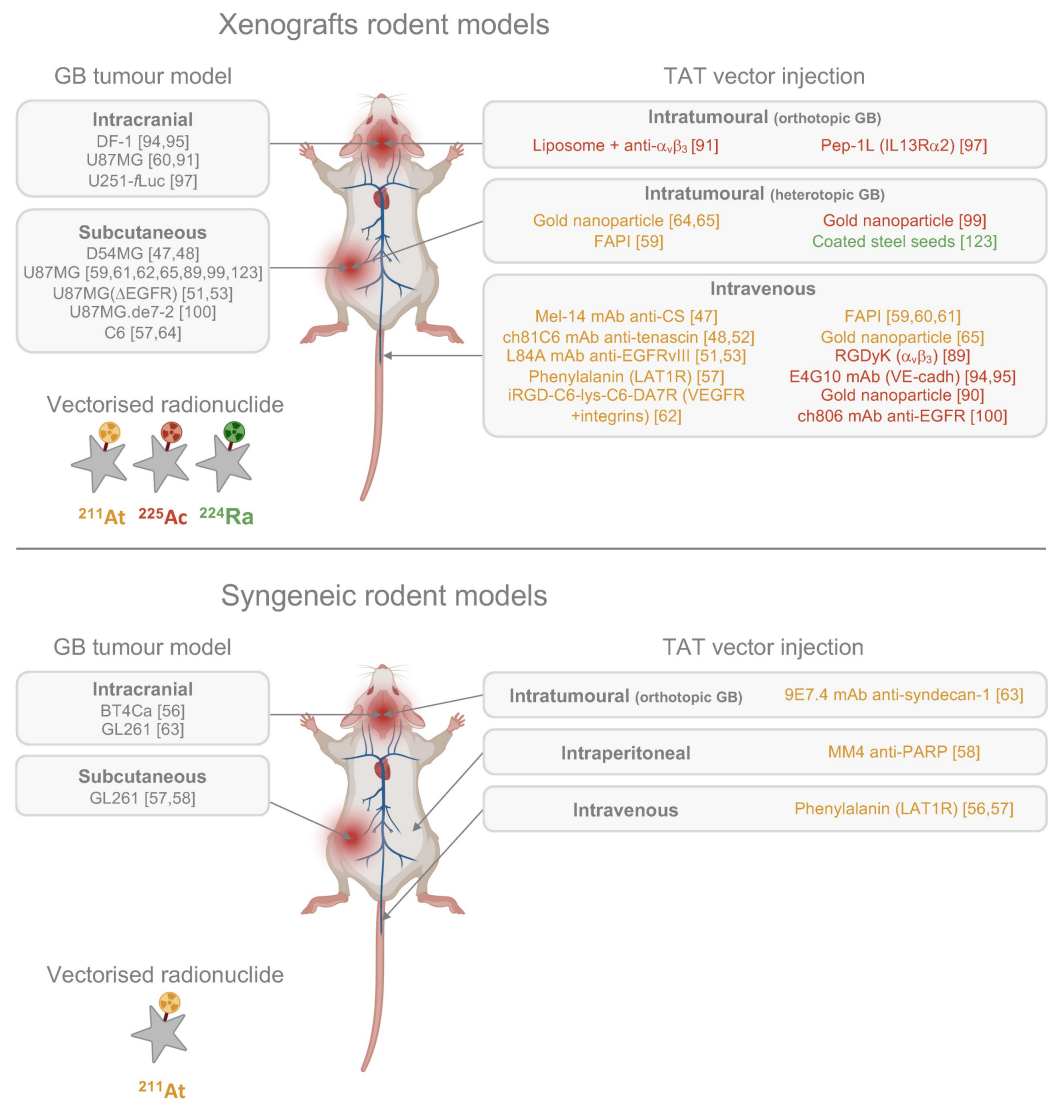
** Current TAT designs and administration routes in preclinical studies for GB.** CS: chondroitin sulfate; EGFR: epidermal growth factor; FAPI: fibroblast activation protein inhibitor; IL13R_α2_: interleukine-13 receptor subunit α2; mAb: monoclonal antibody; PARP: poly(ADP-ribose) polymerase; VEGFR: vascular endothelial growth factor. (Created with Biorender - biorender.com).

*Syngeneic models.* The radiobiological effects of α particles on GB cells, particularly their immunogenic properties, are still not well understood. Syngeneic murine models provide a powerful platform to study these interactions; however, their application in TAT research remains limited (Fig. 3). Among these models, the GL261 murine cell line is commonly used. This well-characterised cell line retains an infiltrative profile and exhibits high tumorigenicity in serum-free media 149. However, GL261 cells have a high mutational load and display strong immunogenicity due to elevated major histocompatibility complex I (MHC-I) expression compared to human GB 150, which may lead to a more robust adaptive immune response following TAT. To better mirror the immune microenvironment of GB, mouse cell lines with lower immunogenicity, such as the SB28 line, are more suitable for studying immune responses post-TAT 150. Lacking detectable CD40 expression, SB28 cells represent a weakly immunogenic GB model, exhibiting therapeutic responses that closely mimic those observed in human GB 151,152. However, this cell line is notably homogeneous and underrepresented in the literature, particularly regarding its histological characteristics and its microenvironment 139. Integrating this cell line into future syngeneic models for TAT could provide valuable insights, particularly regarding the ability of α particles to elicit an adaptive immune response against a poorly immunogenic tumour. In parallel, the finding of genes involved in the initiation and progression of GB tumours led to the generation of specific mice models that may also help to better understand the relevance and efficacy of TAT 153.

*Tumour site.* The location of the tumour injection is crucial in preclinical GB models. Subcutaneous GB models are still widely used due to their ease of execution and the ability to visually monitor tumour progression, but lack presence of BBB, glial and neural cell populations, as well as key GB characteristics such as single-cell invasion, tumour necrosis, microvascular proliferation, and often exhibit a more robust anti-tumoural immunity due to their direct connexion to systemic immunity. Consequently, the TME of subcutaneous models does not adequately represent the clinical reality. This is particularly significant because the TME plays a key role in treatment response, especially to radiotherapy 154. Despite these limitations, Among the 22 preclinical GB studies exploring TAT approaches, 14 utilised ectopic models (Fig. 3). While the results from these studies are promising, it remains difficult to fully assess the efficacy of the treatment.

Orthotopic cerebral grafting, while more technically challenging, is strongly recommended as it faithfully replicates the native GB microenvironment with presence of BBB but also the blood-cerebrospinal fluid (CSF) and brain-CSF barriers, as well as native and patrolling neural and non-neural brain cell populations. These features play a critical role in both the design of TAT protocols (e.g., dose, administration method, vector selection) and the biological responses it induces. Orthotopic models also allow for advanced interventions, such as tumour resections or rechallenging surviving animals 63,155,156. In orthotopic grafting, ensuring accurate tumour injection coordinates is particularly important. The striatum is the preferred location, with careful attention to avoid cellular infiltration into the adjacent ventricle 157,158. The review by Assi *et al.* remains a valuable reference for developing stereotaxic models in rats and mice 159.

*TAT administration route*. Preclinical studies have predominantly employed intravenous delivery of TAT, with intracranial injections being less common (Fig. 3). The success of TAT depends on the precise interplay between the radionuclide, the vector, and the target, and the choice between intravenous or intracranial administration is pivotal.

The intravenous delivery requires injecting a high activity of the α-emitter coupled with a high concentration of the targeting vector to compensate for the systemic circulation of the TAT. However, it poses a significant risk of off-target toxicity of the radionuclide before reaching the tumour. Achieving specific targeting and ensuring the radionuclide predominantly accumulates in the tumour is essential for success. Intravenous delivery is less invasive, as it avoids the need for additional surgery, making it an attractive option for patients with GB who cannot undergo surgical resection. Nonetheless, this approach must overcome the challenges posed by the BBB, as its integrity can be highly heterogeneous in patients 160. Furthermore, poorly vascularised tumour regions and isolated infiltrating cells are at great risk of evading the therapeutic effects of TAT due to its reliance on blood circulation for distribution.

The intracranial administration, on the other hand, bypasses the BBB, offering a direct route to the tumour. It can be integrated with surgical resection, taking advantage of the resection cavity for TAT administration, or it can be injected directly into the tumour. Cordier *et al.* demonstrated in 2010 that this method, when used as a primary therapeutic approach, was feasible and allowed for a better tumour delineation post-TAT, which could facilitate subsequent surgical resection 119. Future research should explore these methods and consider using CED, which offers direct drug delivery advantages by ensuring a continuous, positive-pressure micro-infusion of the desired agents through the target tissues *via* principles of bulk flow. Thus, by applying a pressure gradient in place of a concentration gradient, considerations about the molecular weight and diffusivity of the therapeutic agent are bypassed. This facilitates the homogeneous administration of low concentrations of drug to treat a specific brain region, while optimising its intratumoural volume distribution 161,162. Intratumoural retention is crucial for the success of TAT, and it is influenced by the properties of the vector and the selection of a therapeutic target specific to GB. For instance, using a mAb for intratumoural delivery might enhance retention due to its large size (~150 kDa) and prolonged half-life in tissues.

*Dosimetry*. Radiation dosimetry provides a method for standardising and comparing the efficacy of different radiation-based therapies. As outlined in the MIRD Pamphlet No. 22, accurate dosimetry for α-particle emitters requires detailed knowledge of activity distribution over time at the cellular and subcellular levels, supported by precise geometric modelling. Simplified spherical models are effective for *in vitro* studies, but *in vivo* and clinical applications demand complex 3D representations, such as spheroids or biopsy-derived geometries. Microdosimetry is essential due to the high LET and localised effects of α-particles, which can induce significant biological impacts from a single nuclear traversal 31. From the onset of investigations into α-emitters, dosimetric considerations have been integral to understanding their therapeutic potential.

Early studies in the 1990s on ^211^At in murine subcutaneous GB models showed an absorbed dose in the tumour of 1.9 Gy after intravenous injection of an activity of 37 kBq of ^211^At-labelled Mel-14 (Fab')_2_, representing five times the dose achieved with a non-targeted antibody, and 15 times the tumour dose reported previously for ^131^I-Mel-14 47. In 1997, Zalutsky *et al.* estimated absorbed doses for humans following intravenous injection of 74 kBq of ^211^At-81C6 in a mouse model of GB, reporting 2-3 mSv/MBq for most organs, with the highest dose being 32.9 mSv/MBq to bone surfaces. Direct CSF injection reduced these doses by three orders of magnitude, highlighting the potential of locoregional TAT to minimise systemic toxicity 48. Studies on the biodistribution of free ^211^At in nude mice and Sprague rats revealed its highest uptake in the thyroid gland, lungs, spleen, and stomach. Additionally, ^211^At exhibited higher activity concentrations in extrathyroidal organs compared to radioiodide 163-165. Considering these differences, the use of ^131^I or ^125^I is still a reliable approach for initial estimations of the biodistribution and dosimetry of ^211^At. Dosimetry of ^211^At-labelled 9E7.4 mAb was evaluated by Roncali *et al.* using ^125^I as a reference radionuclide. The brain absorbed dose after brain intratumoural injection of 100 kBq of ^211^At-9E7.4 was estimated at 4.35 ± 0.49 Gy, compared to 2.78 ± 0.42 Gy for the ^211^At-labelled isotype control. These doses correspond to 43.5 ± 4.9 Gy/MBq and 27.8 ± 4.2 Gy/MBq, respectively. 63.

Regarding ^225^Ac, Pandya *et al.* demonstrated in a subcutaneous mouse GB model that radioactivity cleared more slowly from the tumour compared to the kidneys following intravenous administration of 700 kBq of ^225^Ac-RGDyK, with absorbed doses calculated at 0.288 Gy in the tumour and 0.301 Gy in the kidneys 89. In an orthotopic mouse GB model, Behling *et al.* reported a tumour absorbed dose of 24.4 ± 4.8 Gy/MBq, which was 7.7 times higher than the dose in the healthy brain (3.1 ± 0.8 Gy/MBq), after intravenous injection of 11.1 kBq of ^225^Ac-E4G10 95. More recently, in a subcutaneous mouse GB model, Wichmann *et al.* observed absorbed doses of 40.66 Gy in U87MGde2-7 tumours and 29.47 Gy in DiFi tumours following intravenous administration of 18.5 kBq of ^225^Ac-ch806 100.

To date, limited data is available in the recent literature regarding dosimetry in TAT for GB studies. As recommended by Tronchin *et al.*, three key points should be considered when performing dosimetry in TAT: (1) Generating accurate biodistribution data, as direct imaging of α-emitters is limited and often requires surrogate radionuclides, blood/faecal sampling, or animal studies. (2) Tracking the migration of free daughter radionuclides since the high decay energy of α-emitters can disrupt their bond with the targeting vector. (3) Performing microdosimetry to assess non-uniform dose distribution and biological effects at the cellular or tissue level, given the short path length and heterogeneous distribution of α-emitters 166.

*Model standardisation*. To improve the efficacy and reliability of TAT for GB, standardising *in vivo* models is crucial. Our recommendations focus on four key aspects:

**1. Adopting orthotopic models.** We strongly advocate for the adoption of orthotopic GB models and the gradual discontinuation of subcutaneous models, which provide limited translational relevance. Syngeneic models should be prioritised, as they remain underexplored in TAT for GB, while allogeneic models should increasingly incorporate PDXs. Although implementing these models is technically challenging and the heterogeneity among PDXs demands greater representation in the literature, doing so will provide a more comprehensive understanding of TAT effects across different patient profiles.

**2. Integrating *in vivo* imaging.** *In vivo* imaging should be a cornerstone of TAT studies. MRI is recommended for survival studies, particularly T1 and T2-FLAIR sequences, with DCE-MRI for monitoring BBB integrity when possible. Quantitative imaging of TAT is also highly encouraged, either through SPECT/CT directly using the radionuclide of interest (e.g., ^225^Ac) or PET/CT with radiolabelled vectors. These techniques provide valuable insights into therapy efficacy and biological responses.

**3. Expanding dosimetry studies.** Quantitative imaging lays the groundwork for integrating dosimetry studies, which are severely lacking in recent TAT research. Calculating absorbed doses in target cells and normal tissues is critical, with digital autoradiography offering a promising approach for detailed assessments. These data will inform optimisation of dosing protocols and improve safety profiles.

**4. Incorporating standard of care.** The inclusion of standard of care treatments into preclinical models is essential for clinical relevance. Resection surgery should be incorporated to study therapeutic modalities applicable to varying resection quality. Additionally, integrating the Stupp regimen into these models would allow researchers to evaluate potential synergies or antagonisms between TAT, chemotherapy, and external radiotherapy. In this context, the murine GB model developed by Le Reste *et al.*, replicating the standard-of-care protocol with surgical resection followed by the Stupp regimen, could be highly useful 155. Introducing TAT during the surgical phase of such protocols, rather than as a standalone therapy, could offer insights into its compatibility with standard treatments. Despite intracranial treatments showing safety and feasibility in clinical trials, they remain underrepresented in preclinical studies of TAT for GB. Addressing these gaps will align preclinical research more closely with clinical realities, facilitating the transition from experimental studies to therapeutic applications and ultimately improving patient outcomes.

### Targeting innovations and emerging vectors

**Figure 4:**
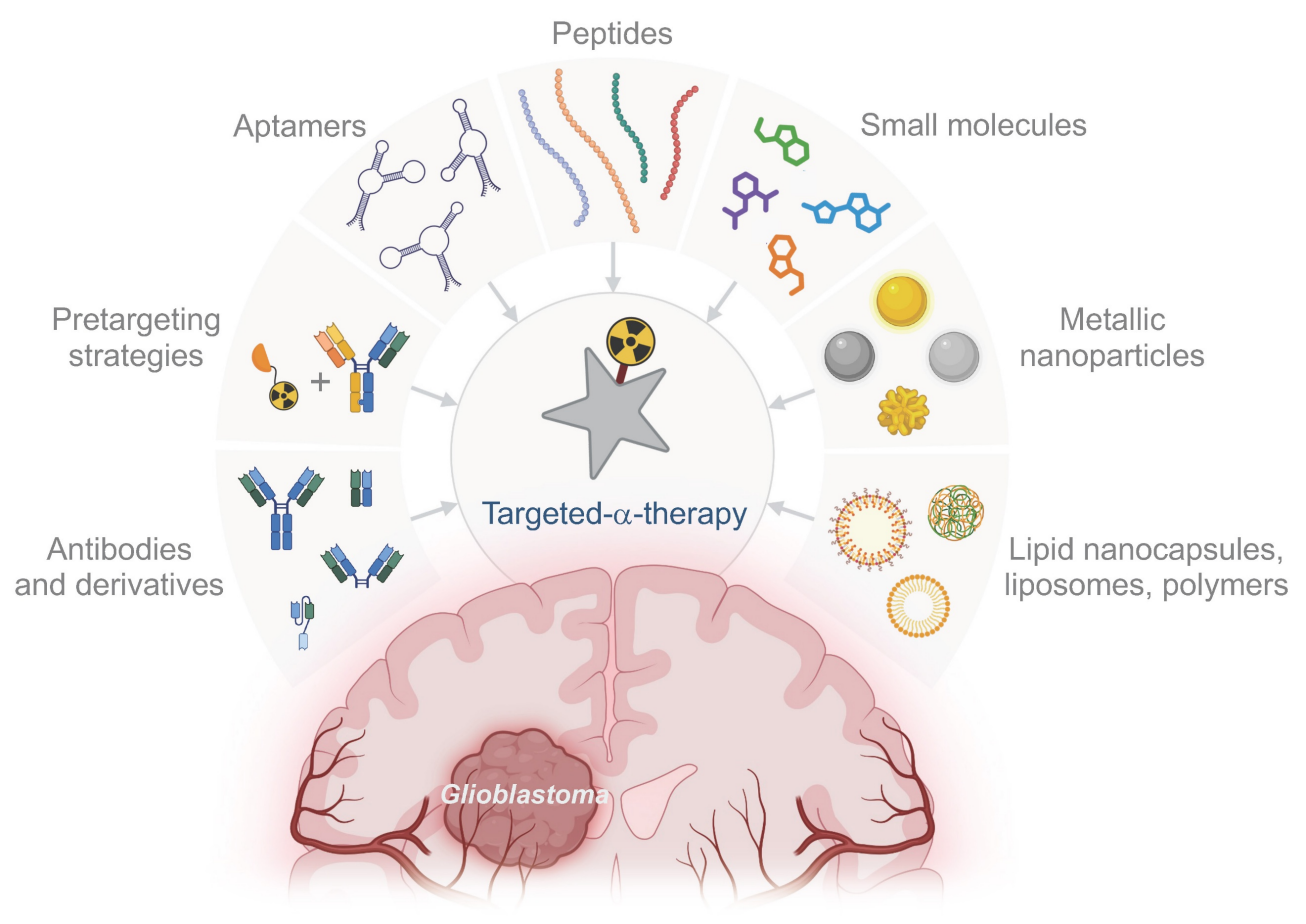
** Potential vectors for the design of future TATs in GB.** (Created with Biorender - biorender.com).

The primary challenge in treating GB is its significant intratumoural heterogeneity, which challenges the selection of effective therapeutic targets. The goal of targeted therapy is to eliminate tumour cells, making the timing of therapeutic administration crucial to effectively target invasive cells before they become unreachable. Current preclinical studies of TAT for GB use various vectors, including mAbs, peptides, non-peptidic small molecules, and AuNPs, for both local and systemic strategies (Fig. 3). However, many promising vectors remain underexplored (Fig. 4). Beyond considerations of cost and ease of production, the choice of vector must align with the overall TAT design, considering the physicochemical properties of the radionuclide - particularly its half-life - the biological target and its expression on tumour and healthy cells, as well as the administration route chosen to reach the GB, with BBB crossing posing an additional challenge in cases of systemic administration.

*Antibodies and derivates*. mAbs have shown clinical efficacy in TRT for GB with both β^-^ and α-emitting radionuclides. Some FDA-approved TRT treatments, such as Zevalin^®^ and Bexxar^®^, are indeed based on anti-CD20 mAbs 26,27. mAbs, due to their large molecular weight, have low tissue diffusivity and slow clearance. These properties can be advantageous for locoregional administration of TRT when combined with a short-lived radionuclide, ensuring prolonged retention within the tumour throughout the decay process. However, these same characteristics can be challenging for systemic TRT administration, especially when using radionuclides with long half-lives, as this can increase the risk of off-target toxicity.

In TAT for GB based on mAbs, only the targeting of tenascin-C 48,52,55, EGFRvIII 51,53, VE-cadherin 94,95, and more recently syndecan-1 63, has been extensively tested. However, historical targets used in TRT could provide promising new opportunities, given the positive outcomes seen with some of these approaches. For example, DNA-Histone H1, an intracellular antigen expressed in the necrotic core of tumour cells 167, has shown encouraging improvements in the lifespan of patients when labelled with ^131^I 168,169. Similarly, the C-X-C chemokine receptor type 4 (CXCR4), a receptor involved in tumour survival, proliferation, and migration 170-172, has demonstrated improved survival in a GB mouse model using a TRT approach with lipid nanocapsules loaded with rhenium-188 (^188^Re) targeting CXCR4 173.

Reducing the size of the vector can enhance diffusivity clearance and access to difficult-to-reach epitopes. As a result, various mAbs derivatives have been developed. Among these, F(ab) and F(ab')_2_ retain one or two variable regions of the mAb, respectively, while lacking the constant Fc region, preserving their binding affinity. Additionally, synthetic scaffolds such as monobodies, nanobodies, affibodies, anticalins, and designed ankyrin repeat proteins (DARPins) offer high affinity and selectivity similar to mAbs but are generally smaller, typically weighing less than 10 kDa. These modular scaffolds also allow for the creation of bispecific molecules, which is particularly advantageous for pretargeting strategies in TAT 174.

*Pretargeting*. Pretargeting is a method that aims to reduce off-target radiation by first delivering a targeting agent to the tumour, followed by the introduction of the radionuclide. The original pretargeting method used a streptavidin-conjugated antibody without a radiolabel, followed by a radiolabelled biotin injection. This approach typically involves administering a bispecific antibody that binds to the therapeutic target, which is later followed by a radiolabelled bivalent hapten peptide 175. Regarding intravenous administration of TRT for GB, pretargeting offers a promising approach, combining the tumour-specific targeting of mAbs with the rapid clearance of radiolabelled small molecules. This allows for a higher tumour dose while reducing radiation to normal tissues compared to directly radiolabelled mAbs. In GB studies, pretargeting strategies using the biotin-streptavidin interaction 176 or targeting fibronectin 177 have been explored with ^131^I. However, challenges include poor uptake of the mAb, dose-limiting toxicities, and the potential for patients to develop antidrug antibodies. Given the high energy levels of α-emitters, pretargeting could be particularly advantageous for TAT. Recently, a three-step pretargeting approach using ^225^Ac was tested to target human epidermal growth factor receptor 2 (HER2) in a murine ovarian cancer model. The method involved an intraperitoneal injection of a bispecific antibody targeting HER2 and DOTA, followed by an intravenous clearing agent, and then the ^225^Ac-labelled hapten. This approach resulted in extended survival with minimal toxicity 178.

*Aptamers.* Often described as “chemical antibodies,” aptamers are short RNA or DNA oligonucleotides capable of binding specific targets with high affinity and remarkable selectivity. These molecules fold into unique three-dimensional structures, enabling them to recognise and bind to their targets similarly to antibodies. Aptamers are generated using the SELEX (Systematic Evolution of Ligands by EXponential enrichment) process, which iteratively selects oligonucleotides from a library for optimal binding to the target. This selection process enhances the specificity and affinity of the aptamers. Unlike antibodies, aptamers offer several advantages, including greater stability, minimal batch-to-batch variation, and reduced toxicity and immunogenicity. Their small size allows for better tissue penetration and access to epitopes that larger antibodies might not reach, making them particularly promising for diagnostic imaging applications. Therefore, they represent interesting candidates to design TAT aimed at crossing the BBB after intravenous administration 179.

The first radiolabelled aptamer, TTA1, was synthesised in 2006 and targets tenascin-C, a protein found in various solid tumours, including GB. This aptamer demonstrated effective tumour uptake and diffusion in GB models following intravenous administration, with significant localisation in the tumour within 3 h and rapid clearance from the kidneys and liver 180. Since then, numerous aptamers have been selected against GB through the SELEX process, and the recent review by Doherty *et al.* highlights the most promising avenues 181. Notably, the AS1411 aptamer is closest to clinical application, having completed a Phase I trial in patients with progressive metastatic cancer and a Phase II trial in patients with renal cell carcinoma. However, results remain unsatisfactory: in the first trial, 8 out of 17 patients demonstrated stable disease 2 months post-treatment, while in the Phase II trial, only 1 out of 35 patients showed a strong and durable response to treatment 182-184.

Regarding integration into TRT strategies, the U2 aptamer was developed to target U87-EGFRvIII cells. This aptamer binds effectively to these cells, inhibiting their proliferation, migration, and invasion, while also impacting downstream signalling pathways. Additionally, U2 enhanced the radiosensitivity of U87-EGFRvIII cells *in vitro* and exhibited improved antitumour effects when combined with ^188^Re *in vivo* 185. Radiolabelling of aptamers with α-emitters has not yet been explored *in vivo*; however, it is entirely feasible and could create opportunities for new therapeutic advances, offering a low-cost and highly accessible targeting vector.

*Peptides.* Peptides are small biomolecules typically composed of less than 50 amino acids. They offer several advantages as vectors for TRT, including non-immunogenicity, favourable pharmacokinetics, and straightforward production. While natural peptides exhibit high affinity for their receptors, they are often rapidly degraded, limiting their efficacy in imaging and therapy. However, peptides can be chemically modified to enhance their stability, receptor affinity, and to facilitate radiolabelling. One challenge in this process is that modifications aimed at improving stability and labelling can sometimes alter the properties of the peptide, particularly when essential amino acids are modified or when the chelating agent introduces steric hindrance. Regulatory peptide receptors, many of which are part of the G protein-coupled receptors (GPCRs) superfamily, are overexpressed in various human tumours 186.

Peptide receptor radionuclide therapies (PRRT) have shown significant clinical promise. Somatostatin, one of the earliest peptides studied in this context, plays a role in regulating the endocrine system, influencing neurotransmission, and modulating cell proliferation through its interaction with the somatostatin receptor (SSTR) family (SSTR1-5). These receptors are overexpressed in GEP-NETs and several other tumour types, including GB 187. The most notable therapeutic success with somatostatin is the NETTER-1 clinical trial, which led to the approval of ^177^Lu-DOTATE (Lutathera^®^) in the USA and Europe for targeting SSTRs in patients with neuroendocrine tumours 28. The safety and efficacy of this treatment were further confirmed in the recent NETTER-2 Phase III clinical trial (NCT03972488) 188. Building on this success, additional peptide vectors are being explored for cancer therapy, with SSTR targeting in GB showing promise. Preclinical developments in PRRT with α-emitters have included targeting IL13R_α2_ using ^225^Ac-labelled Pep-1L peptide 97, and α_v_β_3_ integrin using ^225^Ac or ^211^At-labelled RGD peptides 62,91. The encouraging clinical results of ^211^At-DOTA-SP targeting NK1R have encouraged further research with this peptide 101,120,121. Another promising target for PRRT could be the CXCR4 receptor. A ^177^Lu-labelled peptide, FC231, is currently under clinical investigation for its dual potential in radiopharmaceutical imaging and therapy. Known as ^177^Lu-Pentixather, it shows promise as a potential treatment for GB 189,190. Additionally, gastrin-releasing peptide receptors (GRPRs) are overexpressed in several cancer types, including GB 191. Ongoing clinical trials targeting GRPR with ^212^Pb-labelled bombesin analogues (NCT05283330, recruiting) could potentially be adapted for GB treatment as well.

*Small molecules.* Small molecules present several advantages over antibody-based radiopharmaceuticals, including lower cost, faster pharmacokinetics, and versatile radiolabelling options.

The discovery of cancer-associated fibroblasts (CAFs) in the GB TME and the expression of FAP in both GB cells and non-malignant stromal cells within the TME, makes FAP an attractive target for radiopharmaceuticals 192,193. FAP-specific agents have shown promising results in early studies, indicating the potential for further clinical evaluation. For instance, Ma *et al.* investigated a ^211^At-labelled FAPi, demonstrating favourable intratumoural retention in a murine GB model, along with significant reductions in tumour volume and extended *in vivo* survival 59.

Another compelling therapeutic target is the PARP enzymes family, known for its overexpression in various tumour cells. PARPs are involved in transferring ADP-ribose to proteins, impacting processes like chromatin modulation, transcription, and DNA repair. PARPs are overexpressed in cancer, and tumours with defective homologous recombination may depend on PARP-mediated DNA repair, making them vulnerable to PARP inhibition 194. PARP inhibitors (PARPi) have unveiled therapeutic potential in preclinical studies in GB mouse models 195. Dabagian *et al.* explored this avenue using ^211^At-labelled MM4 targeting PARP in a GB mouse model, showing an extended progression-free survival 58.

Other promising small molecules that are already effective for diverse cancer types could potentially be repurposed for TAT in GB. PSMA, an antigenic glycoprotein initially associated with prostate cancer, is found to be overexpressed in GB, making it as a promising therapeutic target 196. PSMA-targeting therapies have already gained market approval, with Pluvicto^®^ for the treatment of mCRPC 29, and have demonstrated efficacy with ^177^Lu in GB 197,198. Moreover, the radiolabelling of PSMA with ^211^At has been investigated and could also offer benefits for GB 199-201.

*Nanoparticles.* Nanoparticles (NPs) are proving valuable in TRT for GB, allowing for passive local delivery and, in some cases, enabling crossing of the BBB with systemic administration. Conjugated with antibodies, peptides, or small molecules, NPs provide radionuclide protection and active tumour targeting, ensuring better retention and delivering higher doses to the target tissue.

Encouraging outcomes have been achieved using TRT with β-emitters in *in vivo* GB models. For example, intracranial CED injection of lipid nanocapsules loaded with ^188^Re resulted in 83% long-term survival in a rat GB model by effectively bypassing immunosuppressive barriers 156. Other successes include the locoregional CED administration of metallofullerene labelled with ^177^Lu 202, and liposomes with ^186^Re 203. Iron oxide NPs have also been explored for various GB treatments and can be loaded with radionuclides 204,205, offering potential for adaptation to TAT in future studies.

Regarding α-emitters in GB treatment, only AuNPs have been evaluated *in vivo*, specifically with ^211^At and ^225^Ac in subcutaneous models. Therefore, Kato *et al.* investigated ^211^At-labelled AuNPs across a range of sizes from 120 nm down to 5 nm, observing the most substantial antitumour effect with 5 nm AuNPs 64. Liu *et al.* demonstrated that gold nanostars, with a multibranched star shape providing a high surface area for conjugation with ^211^At, exhibited excellent *in vivo* stability and reduced tumour growth following intratumoural injection 65. Salvanou *et al.* also observed delayed tumour growth after intratumoural injection of 5-9 nm ^225^Ac-labelled AuNPs 99. AuNP are already approved by FDA in some biomedical applications and constitute promising assets for the future of TAT, given their favourable size-to-volume ratio that supports local diffusion and renal clearance, their biocompatibility, and their potential for multimodal imaging.

In other tumour types, ^225^Ac have been tested with various NPs. For example, in human prostate cancer cells, anti-PSMA-targeted liposomes (functionalised with an antibody or an aptamer) loaded with ^225^Ac selectively bound to, internalised, and killed PSMA-expressing cells of rat and human prostate cancer *in vitro* 206. Furthermore, PLGA NPs with ^225^Ac increased cell death in breast cancer cells 207, and polymer nanoparticles loaded with ^225^Ac, administered either intratumourally or intravenously, significantly inhibited tumour growth in murine 4T1 models, with a more favourable response observed through the intratumoural route 208. Additionally, ultrasmall silver telluride NPs loaded with ^212^Pb showed good radiochemical stability and nuclear accumulation in U87MG GB cells, making them a promising candidate for a first *in vivo* TAT study in GB with ^212^Pb 209.

Finally, novel NPs not yet explored in TRT also hold promise for GB. Gregory *et al.* investigated synthetic protein NPs, composed of polymerised human serum albumin with the cell-penetrating peptide iRGD, in a GB mouse model. When loaded with siRNA against signal transducer and activator of transcription 3 (STAT3) and combined with ionising radiation, the systemic administration achieved 87.5% long-term survival, along with memory immunity 210. Thus, such innovative vectors could similarly be adapted for TAT applications.

### Therapeutic combinations

**Figure 5:**
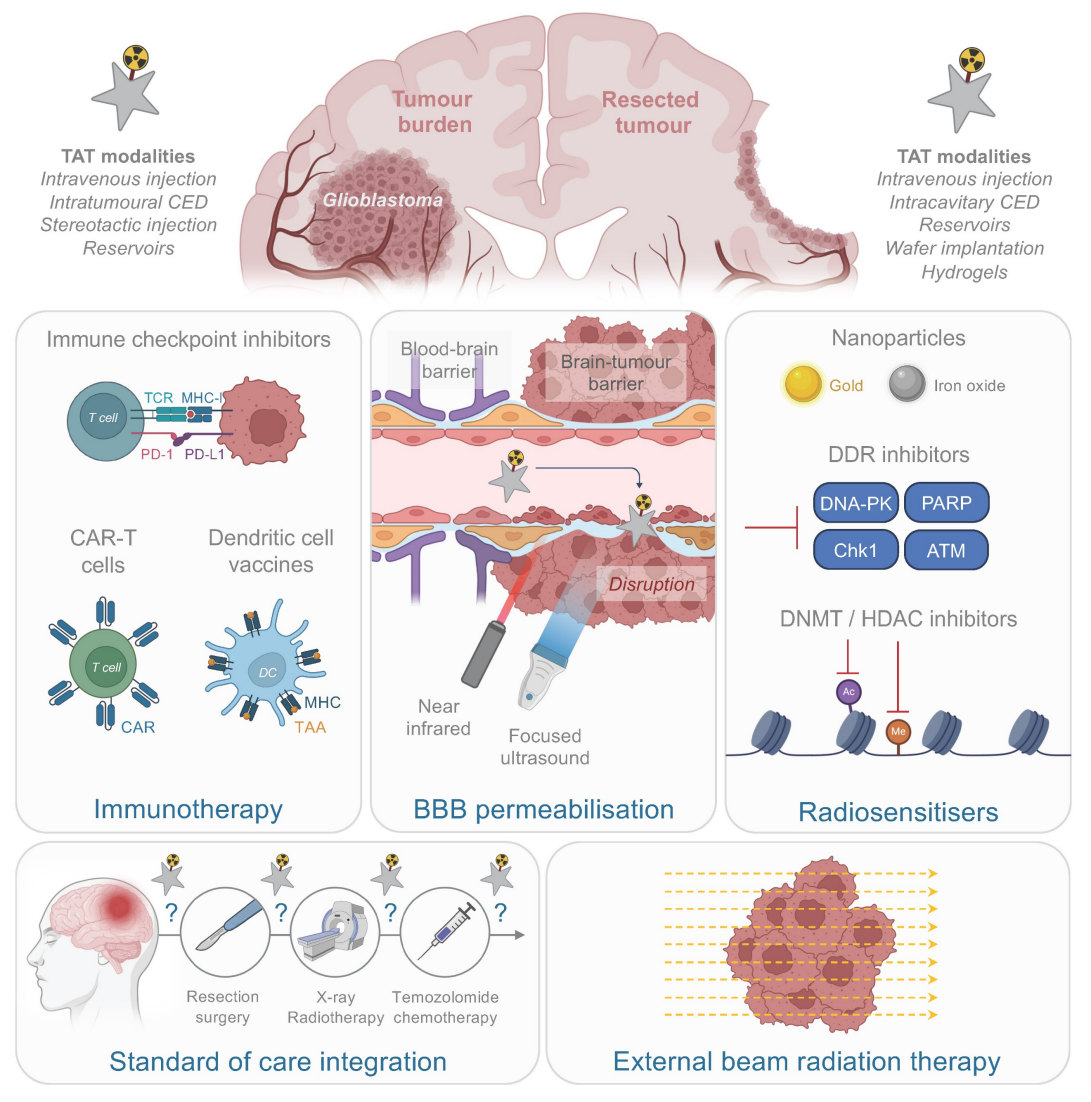
** Potential therapeutic combinations with TAT for GB.** CAR: chimeric antigen receptor; CED: convection-enhanced delivery; DDR: DNA damage response; DNMT: DNA methyltransferase; HDAC: histone deacetylase; MHC: major histocompatibility complex; PD-1: programmed cell death protein 1; PD-L1: programmed death-ligand 1; TAA: tumour-associated antigen; TAT: targeted-α-therapy; TCR: T-cell receptor. (Created with Biorender - biorender.com).

Current TRT methods have achieved some clinical success, but significant improvements are still necessary. Challenges persist due to tumour heterogeneity and the difficulty of delivering radioactive drugs to all tumour cells. As outlined by Obata *et al.*, three key strategies to enhance the effectiveness of TRT should be considered: (1) Increasing the differential cytotoxicity between normal and cancer cells, (2) Enhancing the radiation sensitivity of resistant cancer cells, and (3) Leveraging inflammatory and immune responses to target non-irradiated cells. Combining TAT with other therapeutic approaches could address these issues and improve overall treatment efficacy (Fig. 5) 211.

*Immunotherapy.* The immunogenic effects of α particles offer significant promise for improving therapeutic outcomes and enabling combinations with immunotherapy. Several studies have demonstrated that α particles can modulate tumour-associated antigen presentation, recruit immune cells to the TME, and induce broader immune responses.

Thus, it has been demonstrated that TAT induces the production of damage-associated molecular patterns (DAMPs) both *in vitro* and *in vivo*, including calreticulin, heat shock protein 70 (HSP70), HSP90, and high mobility group box 1 protein (HMGB1) 212-214. This process is accompanied by the release of cytokines and chemokines 214,215, and the activation of the cyclic GMP-AMP synthase (cGAS)- stimulator of interferon genes (STING) signalling pathway, which drives type I interferon production necessary for dendritic cell maturation. For instance, Lejeune *et al.* reported increased transcription of interleukin-6 (IL-6), chemokine CC ligand 20 (CCL-20), and chemokine (C-X-C motif) ligand 10 (CXCL10) *in vitro* following ^227^Th exposure of murine colon adenocarcinoma cells. Similarly, Perrin *et al.* observed elevated levels of IL-2, CCL-5, and interferon-γ along with increased MHC-I expression, in a mouse model of multiple myeloma treated with ^213^Bi-labelled anti-SDC1 antibody 215, and Malamas *et al.* demonstrated that *in vitro* exposure of prostate, lung, and breast cancer cells to ^223^Ra dichloride resulted in the surface exposure of DAMPs and MHC-I, rendering tumour cells more susceptible to T cell-mediated lysis 213.

TAT has also been shown to remodel the immune cell populations within the TME. Perrin *et al.* highlighted a decrease in immunosuppressive regulatory CD4^+^ T cells following TAT application in multiple myeloma 215. In colorectal carcinoma, Lejeune *et al.* reported an enhanced dendritic cell migration and CD8^+^ T cell infiltration after TAT 214. Finally, in a melanoma model treated with ^225^Ac, Urbanska *et al.* reported distinct changes in immune cell populations, including naive and activated CD8^+^ T cells, Th1 and regulatory T cells, immature dendritic cells, monocytes, macrophages, and activated natural killer cells 216. Clinical data on TAT-induced immune responses are also available but remain limited. In a study involving 15 patients with prostate cancer, a reduction in CD8^+^ T cells expressing PD-L1 was noted following ^223^Ra irradiation 217. Furthermore, a case report described an abscopal effect in a cutaneous squamous cell carcinoma treated with ^224^Ra-loaded seeds, where distant untreated lesions were eradicated 218.

In GB models, immune modulation following TAT has been highlighted in three studies. Roncali *et al.* demonstrated that a tumour rechallenge in the hemisphere not exposed to TAT elicited immune memory, indicating the establishment of an anti-tumour immune response following locoregional treatment targeting SDC1 63. Behling *et al.* demonstrated that the antivascular ^225^Ac-E4G10 treatment reduced the regulatory T cell population within the TME 94, and Dabagian *et al.* observed modulation of lymphocyte and neutrophil populations following ^211^At-MM4 targeting PARP 58.

To date, only this last study investigated the combination of TAT with immunotherapy in GB. Thus, it was demonstrated that combining ^211^At-MM4 with an anti-programmed cell death protein 1 (PD-1) antibody resulted in significantly extended progression-free survival compared to monotherapy, alongside a notable increase in neutrophil levels four weeks after administration 58. Other tumour models have shown encouraging results but only rely on ICI combination strategies. In their colorectal carcinoma model, Lejeune et al. showed that 227^Th^ TAT combined with anti-programmed death-ligand 1 (PD-L1) increased the number of tumour-free animals 214. In a murine melanoma model, the combination of melanocortin 1 receptor (MC1R)-targeted ^212^Pb-VMT01 with immune checkpoint inhibitors (ICIs; anti-CTLA-4 and anti-PD-1) proved superior in inhibiting tumour growth compared to treatment alone. Mice that had a complete response showed minimal or no tumour regrowth upon rechallenge, suggesting the development of adaptive antitumour immunity 219. Additionally, ^223^Ra bone metastatic prostate cancer models, has reported T-cell activation with combination treatments with anti-PD-L1 and anti-CTLA4 214,220. However, some combinations have not shown superior efficacy compared to monotherapies. For instance, in a melanoma mouse model, melanin-targeted or PD-L1-targeted ^225^Ac-TAT combined with ICIs did not exceed the effectiveness of monotherapies 221. Success of such combinations might also be influenced by treatment scheduling. In a murine melanoma model treated with ^213^Bi-anti-melanin and anti-PD-1, the highest survival rate was observed when ICI administration was interspersed between two TAT injections 222. Future research should focus on optimising the timing of treatment while thoroughly evaluating the potential toxicity of α particles on immune cells. Although an effective dose might minimise systemic effects, it could still damage or deplete the immune cells recruited to the tumour microenvironment. Careful consideration of these factors is essential to ensure that the immunogenic potential of α particles is harnessed without compromising the overall immune response.

Recent breakthroughs in immunotherapy for GB, following years of limited success, have reopened avenues for exploring promising therapeutic combinations. Among these, a notable advancement is the Phase III clinical trial of a dendritic cell vaccine (DCVax-L) combined with the standard of care, which demonstrated a significant extension of overall survival in both primary and recurrent GB patients, with the treatment being well tolerated 223. Similarly, the safety and bioactivity of chimeric antigen receptor (CAR)-T cells targeting EGFR and IL13R_α2_ were confirmed in a recent Phase I clinical trial 224. For a detailed overview of the immunotherapy landscape in GB, the review by Liu *et al.* provides an in-depth analysis of recent advancements 225. To date, ICIs remain the initial focus for combining immunotherapy with TAT 226 but alternative strategies could also enhance the efficacy of α-particle therapies. For instance, it has been shown that α particles, such as ^213^Bi and ^227^Th, can recruit dendritic cells to the TME. This suggests that combining TAT with dendritic cell vaccines may amplify the therapeutic potential of these approaches. Similarly, as α particles promote T cell recruitment into the TME, they could synergise effectively with CAR-T cell therapies. Further, engineering CAR-T cells to target antigens overexpressed in response to TAT or TAT-induced neoantigens offers another promising avenue for exploration. However, significant work remains to fully understand the immunogenic effects of α particles, particularly in GB. Future efforts should prioritise characterising the signals generated by TAT and the immune cells recruited to the TME. Additionally, defining the precise time frame required for the immune response to develop will be crucial to designing effective TAT-immunotherapy combinations.

*BBB disruption.* The BBB serves as a protective barrier between the brain and the systemic bloodstream, limiting the entry of most small molecules and posing significant challenges for targeting GB. It is composed of specialised endothelial cells reinforced by tight and adherens junctions, pericytes, and astrocytic end-feet 18. Although it was long assumed that the BBB was uniformly disrupted in GB patients, recent clinical findings have called this assumption into question 227,228. In GB, disruption of the BBB is primarily driven by hypoxia-induced VEGF expression, leading to disorganised angiogenesis and the formation of immature, permeable blood vessels within the tumour microenvironment 229,230. Clinical imaging of brain tumours relies on MRI using T1-weighted contrast-enhanced sequences and T2-weighted FLAIR volumes 231. T1-weighted imaging can detect gross disruptions in the BBB but lacks the resolution to capture its heterogeneity, which varies between patients and even within different regions of the same tumour. To address this, dynamic contrast-enhanced MRI (DCE-MRI) quantitatively measures contrast agent transport using pharmacokinetic modelling and dynamic imaging to estimate vascular permeability 232,233. Additional studies using advanced MRI techniques and positron-emission tomography (PET) have shown tumour regions extending beyond contrast-enhanced areas on traditional scans 234. These findings, combined with analyses of resected tissue, support the presence of an intact BBB in certain tumour-adjacent regions. Where the BBB is disrupted, it gives rise to the blood-tumour barrier (BTB), which is characterised by abnormal pericyte distribution, detachment of astrocytic end-feet and neurons (displaced by GB cells), and reduced expression of junctional proteins. While the BTB is often described as hyper-permeable, it remains heterogeneous and retains some BBB-like properties 235.

In the case of TAT using short ranges α-emitters, the homogeneity of dose distribution to the tumour is crucial. In systemic TAT, BBB integrity is a key factor in determining the choice of vector and the administered dose. Enhancing BBB permeability uniformly in the tumour could provide two significant advantages. First, facilitating systemic administration by (1) allowing a broader range of biological vectors that would otherwise be unable to cross the BBB, (2) reducing the activity of radionuclide and quantity of vector administered. Second, breaching the BBB could enhance immune cell recruitment to the tumour site following TAT, even when administered locoregionally. α particles are known to be immunogenic, and increased access of the bloodstream to the tumour could amplify the adaptive immune response. In this regard, it has already been demonstrated that transient BBB disruption significantly increases CD4^+^ T-cell infiltration into the brain 236 and enhances the efficacy of immunotherapies for GB 236,237.

Regarding the ability of α particles to influence BBB integrity, ^225^Ac has been shown to permeabilise the BBB *in vivo*, particularly when delivered using α_v_β_3_-targeted liposomes 91 or an anti-VE-cadherin monoclonal antibody 94. The enhanced vascular permeability induced by α particles could be used in a systemic dose-fractionation protocol, where the first dose primes the BBB, thereby enhancing the effectiveness of subsequent doses, or potentially allowing for dose reductions due to increased permeability. Another approach could involve a combined administration strategy, with a first locoregional dose administered immediately after surgical resection to enhance permeability, followed by a systemic dose to increase therapeutic efficacy.

Thus, external stimulation strategies provide a potential avenue to breach the BBB in the context of TAT. Light is capable of reversibly disrupting the BBB, notably through the use of laser beams. Among light-based methods, near-infrared (NIR) light can penetrate deep into tissues to modulate BBB permeability in a transient way 238. Recently, Cai *et al.* treated GB-bearing mice using a pulsed laser stimulation of AuNPs targeting tight junctions on the blood vessels to induce a transient disruption of the BBB. This disruption then increased the efficacy of paclitaxel chemotherapy. This protocol led to a reduction in tumour growth, with a significant increase in median survival, up to 50% in a mouse model of GB 239.

Focused ultrasound (FUS) methods have also attracted significant attention for BBB modulation, with recent clinical trials proving the safety of this approach. In a recent Phase I clinical trial, Sonabend *et al*. assessed an implantable device emitting low-intensity ultrasound for the delivery of albumin-bound paclitaxel in patients with GB. Their method involved the administration of low-intensity pulsed ultrasound with simultaneous intravenous microbubble injection 240. While the feasibility of this approach has been shown, it should be noted that, in this context, the timeline for the restoration of the BBB depends on the technology employed and the molecular characteristics of the administered drug. Future pharmacokinetic data about drug accumulation and clearance need to be addressed in subsequent research. Another Phase I/II clinical trial evaluated an implantable ultrasound device designed to transiently open the BBB prior to carboplatin chemotherapy. While the study showed an improved control of tumour growth, these effects did not result in a significant improvement in progression-free survival in patients 241, and further investigations in a larger study is ongoing (NCT05902169, recruiting). The ongoing Phase II Sonofirst study (NCT04614493) is also exploring FUS in combination with the Stupp regimen for patients with GB. This approach may improve therapeutic efficacy while reducing systemic toxicity. Sharma *et al.* recently reviewed this concept, noting that ultrasound-stimulated microbubbles can optimise radiation effects and potentially trigger an anti-tumour response 242.

*Radiosensitisers*. Radiosensitisation of GB is a developing field, with research focusing on enhancing tumour cell sensitivity to radiation. Gold and iron oxide NPs have shown potential in augmenting the radiosensitisation of GB cells *in vitro* 243,244. AuNPs, specifically, have been explored for TAT using radionuclides like ^211^At 64,65 and ^225^Ac 99, although their progress has not yet surpassed other strategies.

A promising approach to increase tumour radiosensitivity involves targeting the DNA damage response (DDR). DDR encompasses pathways that repair DNA damage, ensuring genomic stability. Defects in DDR machinery can lead to genome instability, a hallmark of cancer, which results in increased mutational burden and promotes tumorigenesis. Tumour cells rely on DDR mechanisms to survive exposure to genotoxic agents like chemotherapy and radiotherapy. Overexpression of DDR regulatory proteins aids in DNA repair, thus facilitating cell survival. Inhibiting these DDR mechanisms can increase tumour cell sensitivity to such agents, making the combination of TAT with DDR inhibitors a potentially effective strategy for tumour eradication.

Key DDR kinases include ataxia telangiectasia mutated (ATM), ataxia telangiectasia and rad3-related (ATR), DNA-dependent protein kinase (DNA-PK), and PARP. ATM plays a crucial role in DDR and cell cycle regulation following DNA damage, particularly double-strand breaks (DSBs). ATR, a serine/threonine kinase, is involved in detecting DNA damage, especially single-strand breaks (SSBs). While SSBs are repaired through mechanisms such as base excision repair, nucleotide excision repair, and mismatch repair, DSBs are primarily repaired through homologous recombination and non-homologous end joining. Aberrant activation of DDR kinases is associated with resistance to genotoxic treatments, making these proteins prime targets to enhance tumour cell sensitivity 245.

Additionally, radiation causes complex DNA damage with multiple non-DSB lesions, called clustered DNA damage. Such complex damages necessitate prolonged activation of the repair system, potentially resulting in incomplete repair and mutation induction 246. The type of DNA damage and the repair mechanisms involved vary based on the type of particle and the energy level they emit. For instance, a recent study demonstrated that high-LET particles generate apurinic/apyrimidinic sites and thymine glycol near DSBs. This triggers the initiation of a specific repair pathway, notably involving DNA polymerase θ 247. Further characterisation of α-particle-induced DNA damage should be of great interest for the design of future TAT and the setting of optimal combination with radiosensitisers.

Clinical trials exploring DDR inhibitors (DDRi) for glioma treatment have been extensively reviewed. In TRT, many DDRi have been radiolabelled with radionuclides such as ^123^I, ^131^I, ^18^F, and ^211^At 248. Notably, Makvandi *et al.* labelled a PARP inhibitor (PARPi) with ^211^At, demonstrating its efficacy in a neuroblastoma mouse model. This labelled PARPi was significantly more effective than talazoparib alone, indicating that cell lethality was largely due to α particle-induced DNA damage rather than pharmacological inhibition of PARP 249. In their review, Everix *et al.* suggested that inhibitors like AZD1390 (ATM inhibitor), Nedisertib (M3814, DNA-PK inhibitor), and Chk1 inhibitors SAR-020106 and MK8776, which possess halogenated aryl structures, could be suitable candidates for combination with TAT 248.

Additionally, targeting epigenetic regulation of DNA, which controls gene activation or silencing, could enhance TRT. Focusing on DNA methyltransferases (DNMT) and histone deacetylases (HDAC) offers another strategy, as epigenetic modifications can influence cancer progression. Many DNMT and HDAC inhibitors have FDA approval, making them promising candidates for combination with TAT 211. Furthermore, recent studies have shown that the use of these inhibitors induces the formation of tumour neoantigens and activates antitumour activity 250,251.

## Conclusion

In the effort to advance effective radiopharmaceuticals and therapeutic combinations into clinical use for GB, progress has faced many challenges, but significant breakthroughs are now being achieved. To date, only one mAb (81C6 anti-tenascin-C) and one peptide (substance P) have entered clinical trial and pilot studies, respectively. The α-emitters currently under investigation include ^211^At, ^225^Ac, and ^213^Bi, which have shown promise but have yet to achieve entirely satisfactory results.

In the preclinical landscape, all studied strategies show efficacy in controlling tumour growth with low toxicity. However, in the context of an orthotopic GB model, only one locoregional approach using ^211^At has resulted in long-term survivors 63. While clinical research increasingly favours locoregional strategies, preclinical studies predominantly focus on systemic interventions. The preclinical characterisation of microenvironmental responses induced *in vivo* by TAT remains poorly understood. However, vascular microenvironment remodelling has been demonstrated with ^225^Ac 94,95, and ^211^At prove its ability to generate a memory immune response in survivors, suggesting the involvement of antitumour immunity following treatment 63.

## Perspectives and challenges of TAT in GB treatment

Future *in vivo* investigations of TAT should prioritise the study of DNA damage and immune responses to better understand the impact of α particles on the TME, optimise therapy design, and explore novel therapeutic combinations. In terms of vectors, mAbs should not be disregarded prematurely, as their distinct properties could enhance the efficacy of locoregional TAT strategies. Meanwhile, aptamers and pretargeting approaches offer promising potential to minimise off-target effects in systemic treatments. Currently, no single approach stands out as a definitive leader in TAT for GB in clinical conditions. Each vector, radionuclide, and target offer distinct potential for clinical development. Given the complex nature of GB, it is imperative to develop strategies that comprehensively address the TME, especially regarding immunosuppressive mechanisms. Targeting critical components within this microenvironment through well-designed TAT and therapeutic combinations could destabilise the GB ecosystem and pave the way for its complete eradication. The increased production of α-emitters, coupled with advancements in radiochemistry and the development of compact vectors, suggests a promising future.

To enhance the relevance and translational impact of preclinical research, standardisation of *in vivo* models is essential. Efforts should shift away from subcutaneous GB models towards orthotopic models that more closely mimic human GB. For allogeneic studies, these should ideally use genetically relevant GB cell lines or PDXs. Syngeneic models, which remain underrepresented, should also be explored to deepen our understanding of the immunogenicity of α particles in the context of GB. Systematic imaging and dosimetry studies should also be integrated into research protocols. Furthermore, monitoring BBB integrity in future models could help to refine TAT strategies for both systemic and locoregional applications. Finally, benchmarking TAT against existing standards - either as a standalone adjuvant therapy or in combination with the Stupp regimen - will be critical in determining its therapeutic value and guiding clinical translation.

Economic and logistical challenges must also be addressed to facilitate the broader implementation of TAT. A key obstacle is the limited supply of radionuclides. Efforts to scale production are underway, with new technologies and facilities being developed to address these limitations. As reviewed by Ostuni *et al.*, the global radiopharmaceutical therapy market was valued at $7.78 billion in 2021 and is expected to reach $13.07 billion by 2030, with TAT representing a major growth area due to its clinical potential. Specifically, the α radionuclide market was valued at $672.3 million in 2020 and is projected to grow to $5.2 billion by 2027. The increasing attractiveness of TAT is evidenced by the emergence of new companies and the involvement of major industry players such as Bayer and Novartis. The choice of production and distribution models is also critical to ensuring a reliable supply chain. Centralised production, while providing consistent quality, carries risks of disruption that could impact clinical availability. In contrast, decentralised models offer greater resilience and faster access to radionuclides but require regulatory frameworks to ensure quality and safety.

Addressing these challenges will require sustained investments in isotope production, infrastructure, and delivery systems. A combination of innovative technologies and strategic production networks will be key to overcoming current limitations, enabling the integration of TAT into clinical practice 252.

## Figures and Tables

**Table 1:**
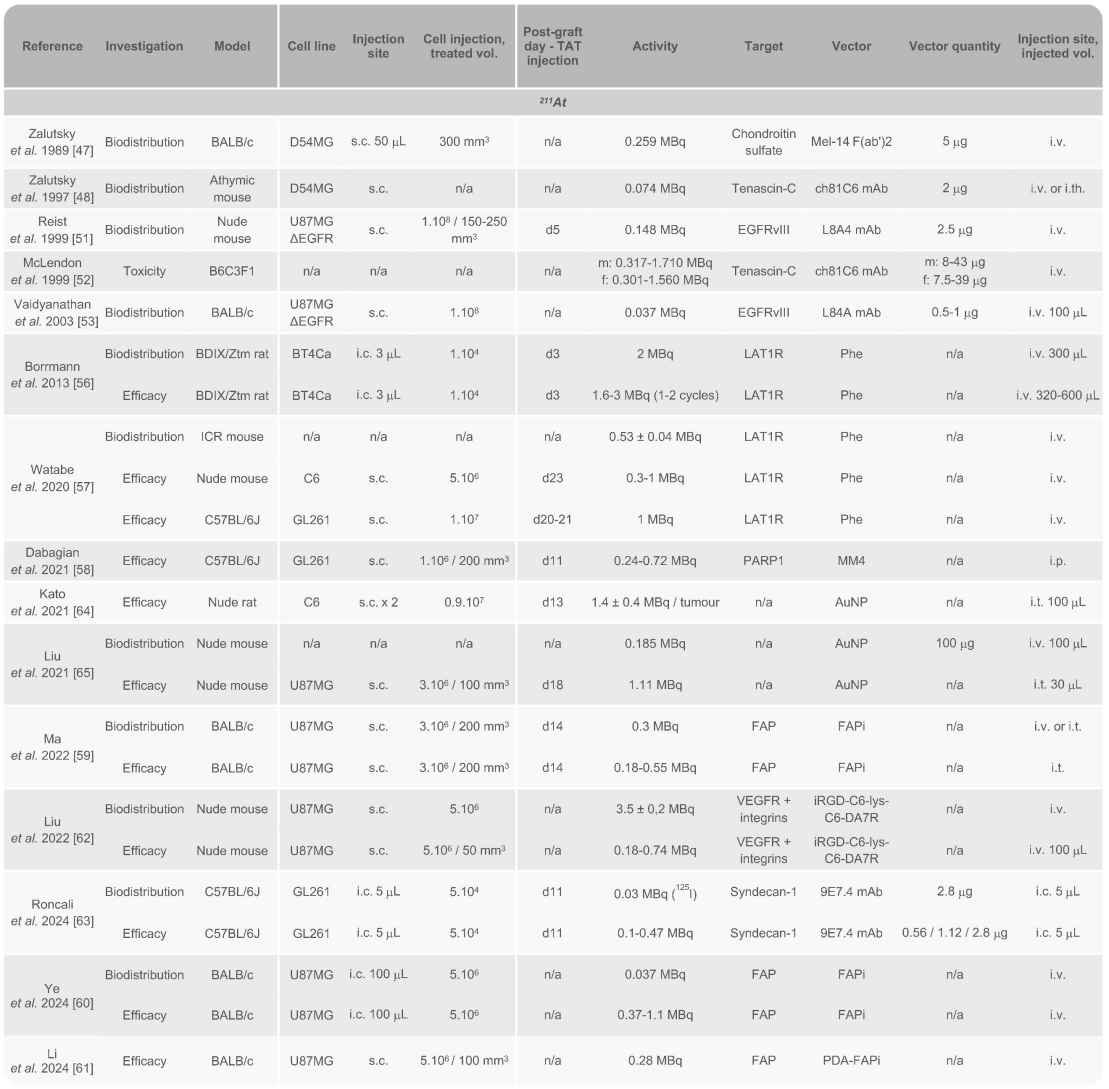
Preclinical studies of targeted-α-therapy using ^211^At in GB *in vivo* models

AuNP: gold nanoparticle; EGFR: epidermal growth factor receptor; FAPi: fibroblast activation protein inhibitor; f: female; s.c.: subcutaneous; i.c.: intracranial; i.t.: intratumoural; i.v.: intravenous; m: male; mAb: monoclonal antibody; PARP1: poly(ADP-ribose) polymerase 1; PDA: polydopamine; PD-L1: programmed death-ligand 1; Phe: phenylalanine; VEGFR: vascular endothelial growth factor receptor.

**Table 2:**
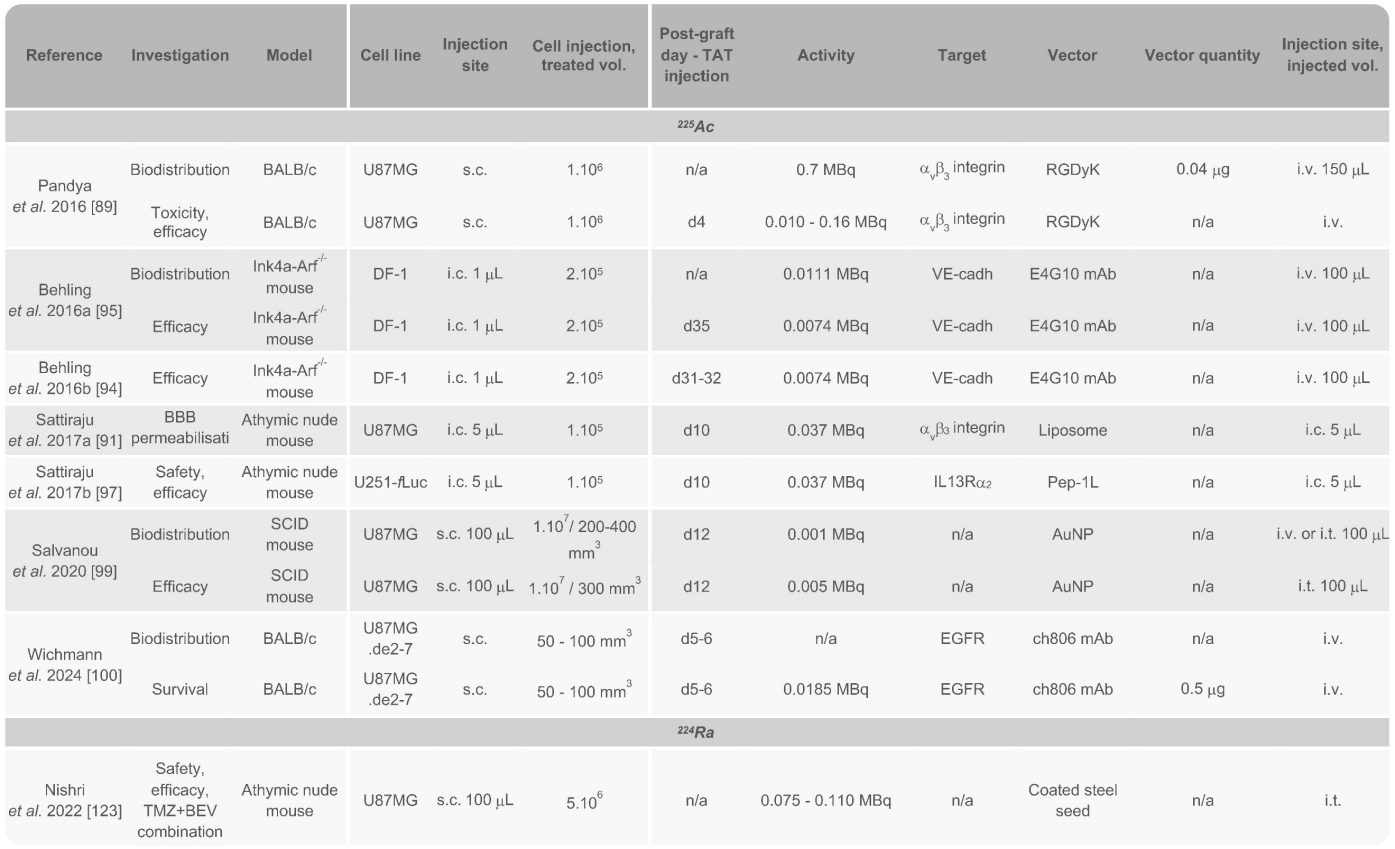
Preclinical studies of targeted-α-therapy using ^225^Ac and ^224^Ra in GB *in vivo* models

AuNP: gold nanoparticle; BBB: blood-brain barrier; BEV: bevacizumab; EGFR: epidermal growth factor receptor; i.c.: intracranial; IL13R_α2_: interleukine-13 receptor subunit α2; i.t.: intratumoural; i.v.: intravenous; mAb: monoclonal antibody; s.c.: subcutaneous; TMZ: temozolomide; VE-cadh: vascular endothelial cadherin.

**Table 3:**
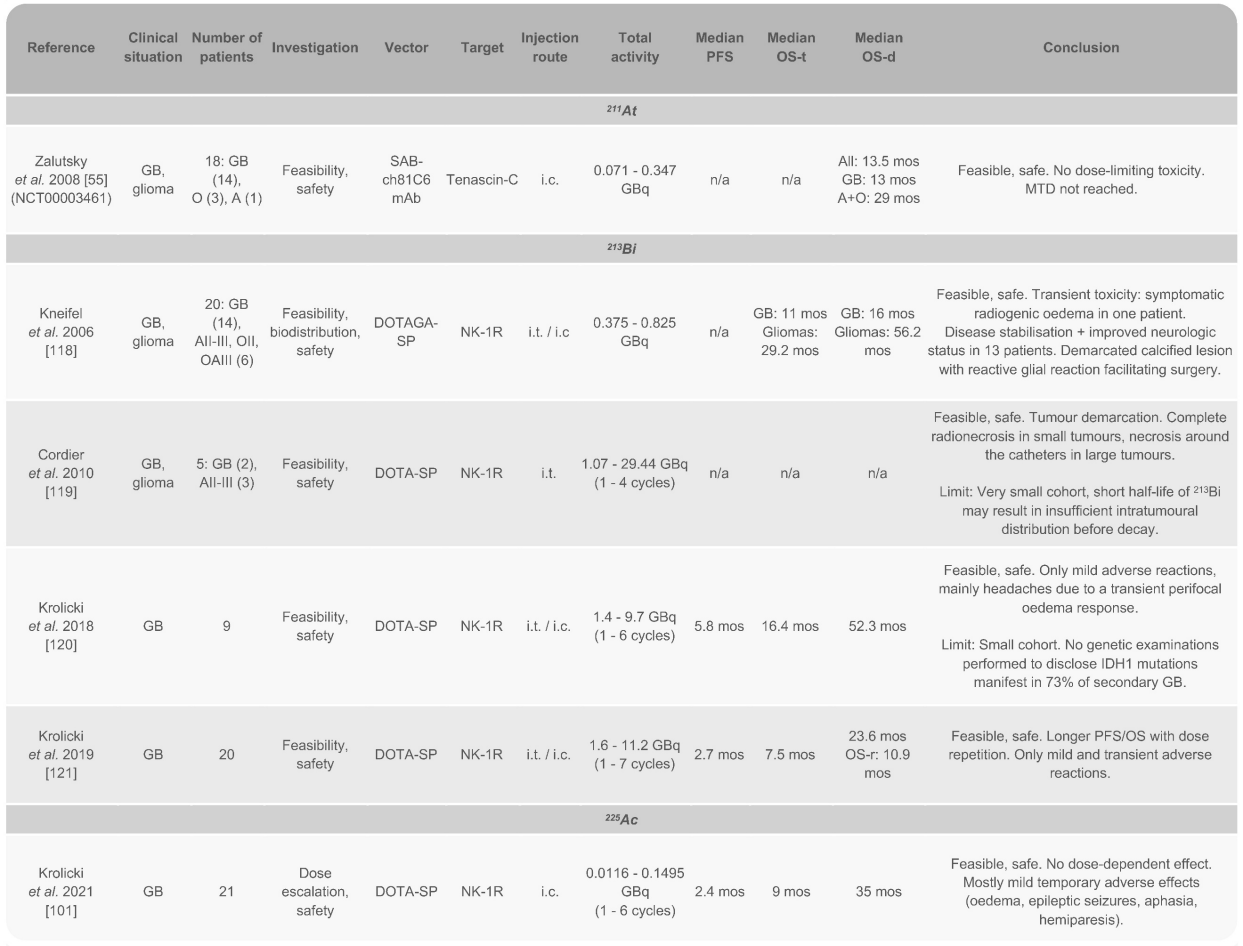
Completed clinical trials and pilot studies in high-grade gliomas treated with TAT

A: astrocytoma; GB: glioblastoma; i.c.: intracranial; i.t.: intratumoural; mos: months; MTD: maximal tolerated dose; O: oligodendroglioma; OS-d: overall survival from diagnosis; OS-r: overall survival from recurrence; OS-t: overall survival from treatment injection; PFS: progression-free survival; SP: substance P.

## Abbreviations

ATM: ataxia telangiectasia mutated
ATR: ataxia telangiectasia and rad3-related
AuNPs: gold nanoparticles
bFGF: fibroblast growth factor b
BBB: blood-brain barrier
BTB: brain-tumour barrier
CAF: cancer-associated fibroblast
CAR: chimeric antigen receptor
CCL: chemokine CC ligand
CED: convection-enhanced delivery
cGAS: cyclic GMP-AMP synthase
Chk1: checkpoint-kinase 1
CSF: cerebrospinal fluid
CXCL: chemokine (C-X-C motif) ligand
CXCR4: C-X-C chemokine receptor type 4
DARPin: designed ankyrin repeat protein
DCE-MRI: dynamic contrast-enhanced MRI
DDR: DNA damage response
DLL3: delta-like ligand 3
DNA-PK: DNA-dependent protein kinase
DNMT: DNA methyltransferase
DOTA: 1-,4-,7-,10-tetraazcy-clododecane-1,4-,7-,10-tetra acetic acid
DSB: double-strand break
EGFR: epidermal growth factor receptor
FAP: fibroblast activation protein
FDA: food and drug administration
FLAIR: Fluid-attenuated inversion recovery
FUS: focus ultrasound
GB: glioblastoma
GEP-NET: gastroenteropancreatic neuroendocrine tumour
GRPR: gastrin-releasing peptide receptor
GPCR: G protein-coupled receptor
GSLC: glioblastoma stem-like cell
HDAC: histone deacetylase
HER2: human epidermal growth factor receptor
HMGB1: high mobility group box 1 protein
HSP: heat shock protein
ICI: immune checkpoint inhibitor
IL13R_α2_: interleukine-13 receptor subunit α2
LAT1R: L-type amino acid transporter 1 receptor
LD_10_: lethal dose for 10% of animals
LET: linear energy transfer
mAb: monoclonal antibody
MC1R: melanocortin 1 receptor
mCRPC: metastatic castration-resistant prostate cancer
MHC-I: major histocompatibility complex I
MRI: magnetic resonance imaging
MTD: maximum tolerated dose
NIR: near-infrared
NK_1_R: neurokinin type 1 receptor
PARP: poly(ADP-ribose) polymerase
PD-1: programmed cell death protein 1
PD-L1: programmed death-ligand 1
PDX: patient-derived xenograft
PRRT: peptide receptor radionuclide therapy
PSMA: prostate-specific membrane antigen
ROS: reactive oxygen species
SDC1: syndecan-1
SELEX: systematic evolution of ligands by exponential enrichment
SPECT: single photon emission computed tomography
SSB: single-strand break
SSTR: somatostatin receptor
STAT3: signal transducer and activator of transcription 3
STING: stimulator of interferon genes
TAT: targeted-α-therapy
TME: tumour microenvironment
TMZ: temozolomide
TRT: targeted radionuclide therapy
TTFs: tumour-treating fields
VEGF: vascular endothelial growth factor

## References

[1] Louis DN, Perry A, Wesseling P, Brat DJ, Cree IA, Figarella-Branger D (2021). The 2021 WHO classification of tumors of the central nervous system: a summary. Neuro Oncol.

[2] Ostrom QT, Price M, Neff C, Cioffi G, Waite KA, Kruchko C (2023). CBTRUS statistical report: primary brain and other central nervous system tumors diagnosed in the United States in 2016-2020. Neuro Oncol.

[3] Stupp R, Mason WP, van den Bent MJ, Weller M, Fisher B, Taphoorn MJB (2005). Radiotherapy plus concomitant and adjuvant temozolomide for glioblastoma. N Engl J Med.

[4] Gilbert MR, Dignam JJ, Armstrong TS, Wefel JS, Blumenthal DT, Vogelbaum MA (2014). A randomized trial of bevacizumab for newly diagnosed glioblastoma. N Engl J Med.

[5] Chinot OL, Wick W, Mason W, Henriksson R, Saran F, Nishikawa R (2014). Bevacizumab plus radiotherapy-temozolomide for newly diagnosed glioblastoma. N Engl J Med.

[6] Stupp R, Taillibert S, Kanner A, Read W, Steinberg D, Lhermitte B (2017). Effect of tumor-treating fields plus maintenance temozolomide vs maintenance temozolomide alone on survival in patients with glioblastoma: a randomized clinical trial. JAMA.

[7] Ballo MT, Conlon P, Lavy-Shahaf G, Kinzel A, Vymazal J, Rulseh AM (2023). Association of tumor treating fields (TTFields) therapy with survival in newly diagnosed glioblastoma: a systematic review and meta-analysis. J Neurooncol.

[8] Chen E, Ling AL, Reardon DA, Chiocca EA (2024). Lessons learned from phase 3 trials of immunotherapy for glioblastoma: time for longitudinal sampling?. Neuro Oncol.

[9] Eisenbarth D, Wang YA (2023). Glioblastoma heterogeneity at single cell resolution. Oncogene.

[10] Mathur R, Wang Q, Schupp PG, Nikolic A, Hilz S, Hong C (2024). Glioblastoma evolution and heterogeneity from a 3D whole-tumor perspective. Cell.

[11] Wang X, Sun Q, Wang W, Liu B, Gu Y, Chen L (2023). Decoding key cell sub-populations and molecular alterations in glioblastoma at recurrence by single-cell analysis. Acta Neuropathol Commun.

[12] Sattiraju A, Kang S, Giotti B, Chen Z, Marallano VJ, Brusco C (2023). Hypoxic niches attract and sequester tumor-associated macrophages and cytotoxic T cells and reprogram them for immunosuppression. Immunity.

[13] Li S, Dong L, Pan Z, Yang G (2023). Targeting the neural stem cells in subventricular zone for the treatment of glioblastoma: an update from preclinical evidence to clinical interventions. Stem Cell Res Ther.

[14] Yeo AT, Rawal S, Delcuze B, Christofides A, Atayde A, Strauss L (2022). Single-cell RNA sequencing reveals evolution of immune landscape during glioblastoma progression. Nat Immunol.

[15] Osswald M, Jung E, Sahm F, Solecki O, Venkataramani V, Blaes J (2015). Brain tumour cells interconnect to a functional and resistant network. Nature.

[16] Weller M, Cloughesy T, Perry JR, Wick W (2013). Standards of care for treatment of recurrent glioblastoma-are we there yet?. Neuro Oncol.

[17] Lemée JM, Clavreul A, Menei P (2015). Intratumoral heterogeneity in glioblastoma: don't forget the peritumoral brain zone. Neuro Oncol.

[18] Wu D, Chen Q, Chen X, Han F, Chen Z, Wang Y (2023). The blood-brain barrier: structure, regulation, and drug delivery. Signal Transduct Target Ther.

[19] Daguenet E, Louati S, Wozny AS, Vial N, Gras M, Guy JB (2020). Radiation-induced bystander and abscopal effects: important lessons from preclinical models. Br J Cancer.

[20] Bigner DD, Brown MT, Friedman AH, Coleman RE, Akabani G, Friedman HS (1998). Iodine-131-labeled antitenascin monoclonal antibody 81C6 treatment of patients with recurrent malignant gliomas: phase I trial results. J Clin Oncol.

[21] Akabani G, Cokgor I, Coleman RE, González Trotter D, Wong TZ, Friedman HS (2000). Dosimetry and dose-response relationships in newly diagnosed patients with malignant gliomas treated with iodine-131-labeled anti-tenascin monoclonal antibody 81C6 therapy. Int J Radiat Oncol Biol Phys.

[22] Cokgor I, Akabani G, Kuan CT, Friedman HS, Friedman AH, Coleman RE (2000). Phase I trial results of iodine-131-labeled antitenascin monoclonal antibody 81C6 treatment of patients with newly diagnosed malignant gliomas. J Clin Oncol.

[23] Grana C, Chinol M, Robertson C, Mazzetta C, Bartolomei M, De Cicco C (2002). Pretargeted adjuvant radioimmunotherapy with Yttrium-90-biotin in malignant glioma patients: A pilot study. Br J Cancer.

[24] Reardon DA, Akabani G, Coleman RE, Friedman AH, Friedman HS, Herndon JE 2nd (2002). Phase II trial of murine ^131^I-labeled antitenascin monoclonal antibody 81C6 administered into surgically created resection cavities of patients with newly diagnosed malignant gliomas. J Clin Oncol.

[25] Casacó A, López G, García I, Rodríguez JA, Fernández R, Figueredo J (2008). Phase I single-dose study of intracavitary-administered Nimotuzumab labeled with ^188^Re in adult recurrent high-grade glioma. Cancer Biol Ther.

[26] Witzig TE, Gordon LI, Cabanillas F, Czuczman MS, Emmanouilides C, Joyce R (2002). Randomized controlled trial of yttrium-90-labeled ibritumomab tiuxetan radioimmunotherapy versus rituximab immunotherapy for patients with relapsed or refractory low-grade, follicular, or transformed B-cell non-Hodgkin's lymphoma. J Clin Oncol.

[27] Kaminski MS, Zelenetz AD, Press OW, Saleh M, Leonard J, Fehrenbacher L (2001). Pivotal study of iodine I 131 tositumomab for chemotherapy-refractory low-grade or transformed low-grade B-cell non-Hodgkin's lymphomas. J Clin Oncol.

[28] Strosberg J, Wolin E, Chasen B, Kulke M, Bushnell D, Caplin M (2018). Health-related quality of life in patients with progressive midgut neuroendocrine tumors treated with ^177^Lu-dotatate in the phase III NETTER-1 trial. J Clin Oncol.

[29] Sartor O, de Bono J, Chi KN, Fizazi K, Herrmann K, Rahbar K (2021). Lutetium-177-PSMA-617 for metastatic castration-resistant prostate cancer. N Engl J Med.

[30] Marcu L, Bezak E, Allen BJ (2018). Global comparison of targeted alpha vs targeted beta therapy for cancer: *in vitro*, *in vivo* and clinical trials. Crit Rev Oncol Hematol.

[31] Sgouros G, Roeske JC, McDevitt MR, Palm S, Allen BJ, Fisher DR (2010). MIRD Pamphlet No. 22 (abridged): radiobiology and dosimetry of alpha-particle emitters for targeted radionuclide therapy. J Nucl Med.

[32] Wulbrand C, Seidl C, Gaertner FC, Bruchertseifer F, Morgenstern A, Essler M (2013). Alpha-particle emitting ^213^Bi-anti-EGFR immunoconjugates eradicate tumor cells independent of oxygenation. PLoS One.

[33] Parker C, Nilsson S, Heinrich D, Helle SI, O'Sullivan JM, Fosså SD (2013). Alpha emitter radium-223 and survival in metastatic prostate cancer. N Engl J Med.

[34] Radchenko V, Morgenstern A, Jalilian AR, Ramogida CF, Cutler C, Duchemin C (2021). Production and supply of α-particle-emitting radionuclides for targeted α-therapy. J Nucl Med.

[35] Eychenne R, Chérel M, Haddad F, Guérard F, Gestin JF (2021). Overview of the most promising radionuclides for targeted alpha therapy: the 'hopeful eight'. Pharmaceutics.

[36] Tosato M, Favaretto C, Kleynhans J, Burgoyne AR, Gestin JF, van der Meulen NP (2025). Alpha atlas: Mapping global production of α-emitting radionuclides for targeted alpha therapy. Nucl Med Biol.

[37] Jefferson RD, Goans RE, Blain PG, Thomas SHL (2009). Diagnosis and treatment of polonium poisoning. Clin Toxicol (Phila).

[38] Henriksen G, Messelt S, Olsen E, Larsen RH (2001). Optimisation of cyclotron production parameters for the ^209^Bi(α, 2n) ^211^At reaction related to biomedical use of ^211^At. Appl Radiat Isot.

[39] Lindegren S, Albertsson P, Bäck T, Jensen H, Palm S, Aneheim E (2020). Realizing clinical trials with astatine-211: the chemistry infrastructure. Cancer Biother Radiopharm.

[40] Feng Y, Zalutsky MR (2021). Production, purification and availability of ^211^At: near term steps towards global access. Nucl Med Biol.

[41] Vanermen M, Ligeour M, Oliveira MC, Gestin JF, Elvas F, Navarro L (2024). Astatine-211 radiolabelling chemistry: from basics to advanced biological applications. EJNMMI Radiopharm Chem.

[42] Corson DR, MacKenzie KR, Segrè E (1940). Artificially radioactive element 85. Phys Rev.

[43] Angel I, Pilo Kerman O, Rousso-Noori L, Friedmann-Morvinski D (2020). Tenascin C promotes cancer cell plasticity in mesenchymal glioblastoma. Oncogene.

[44] Zagzag D, Shiff B, Jallo GI, Greco MA, Blanco C, Cohen H (2002). Tenascin-C promotes microvascular cell migration and phosphorylation of focal adhesion kinase. Cancer Res.

[45] Frederick L, Wang XY, Eley G, James CD (2000). Diversity and frequency of epidermal growth factor receptor mutations in human glioblastomas. Cancer Res.

[46] An Z, Aksoy O, Zheng T, Fan QW, Weiss WA (2018). Epidermal growth factor receptor and EGFRvIII in glioblastoma: signaling pathways and targeted therapies. Oncogene.

[47] Zalutsky MR, Garg PK, Friedman HS, Bigner DD (1989). Labeling monoclonal antibodies and F(ab')2 fragments with the alpha-particle-emitting nuclide astatine-211: preservation of immunoreactivity and *in vivo* localizing capacity. Proc Natl Acad Sci U S A.

[48] Zalutsky MR, Stabin MG, Larsen RH, Bigner DD (1997). Tissue distribution and radiation dosimetry of astatine-211-labeled chimeric 81C6, an alpha-particle-emitting immunoconjugate. Nucl Med Biol.

[49] Reist CJ, Batra SK, Pegram CN, Bigner DD, Zalutsky MR (1997). *In vitro* and *in vivo* behavior of radiolabeled chimeric anti-EGFRvIII monoclonal antibody: comparison with its murine parent. Nucl Med Biol.

[50] Larsen RH, Akabani G, Welsh P, Zalutsky MR (1998). The cytotoxicity and microdosimetry of astatine-211-labeled chimeric monoclonal antibodies in human glioma and melanoma cells *in vitro*. Radiat Res.

[51] Reist CJ, Foulon CF, Alston K, Bigner DD, Zalutsky MR (1999). Astatine-211 labeling of internalizing anti-EGFRvIII monoclonal antibody using N-succinimidyl 5-[^211^At]astato-3-pyridinecarboxylate. Nucl Med Biol.

[52] McLendon RE, Archer GE, Larsen RH, Akabani G, Bigner DD, Zalutsky MR (1999). Radiotoxicity of systemically administered ^211^At-labeled human/mouse chimeric monoclonal antibody: a long-term survival study with histologic analysis. Int J Radiat Oncol Biol Phys.

[53] Vaidyanathan G, Affleck DJ, Bigner DD, Zalutsky MR (2003). N-succinimidyl 3-[^211^At]astato-4-guanidinomethylbenzoate: An acylation agent for labeling internalizing antibodies with α-particle emitting ^211^At. Nucl Med Biol.

[54] Garg PK, Harrison GL, Zalutsky MR (1990). Comparative tissue distribution in mice of the alpha-emitter ^211^At and ^13I^I as labels of a monoclonal antibody and F(ab')2 fragment. Cancer Res.

[55] Zalutsky MR, Reardon DA, Akabani G, Coleman RE, Friedman AH, Friedman HS (2008). Clinical experience with alpha-particle emitting 211At: treatment of recurrent brain tumor patients with ^211^At-labeled chimeric antitenascin monoclonal antibody 81C6. J Nucl Med.

[56] Borrmann N, Friedrich S, Schwabe K, Hedrich HJ, Krauss JK, Knapp WH (2013). Systemic treatment with 4-^211^Atphenylalanine enhances survival of rats with intracranial glioblastoma. Nuklearmedizin.

[57] Watabe T, Kaneda-Nakashima K, Shirakami Y, Liu Y, Ooe K, Teramoto T (2020). Targeted alpha therapy using astatine (^211^At)-labeled phenylalanine: A preclinical study in glioma bearing mice. Oncotarget.

[58] Dabagian H, Taghvaee T, Martorano P, Martinez D, Samanta M, Watkins CM (2021). PARP targeted alpha-particle therapy enhances response to PD-1 immune-checkpoint blockade in a syngeneic mouse model of glioblastoma. ACS Pharmacol Transl Sci.

[59] Ma H, Li F, Shen G, Pan L, Liu W, Liang R (2022). *In vitro* and *in vivo* evaluation of ^211^At-labeled fibroblast activation protein inhibitor for glioma treatment. Bioorg Med Chem.

[60] Ye T, Yu Y, Qu G, Ma H, Shi S, Ji J (2024). ^211^At radiolabeled APBA-FAPI for enhanced targeted-alpha therapy of glioma. Eur J Med Chem.

[61] Li F, Ma H, Luo H, Shen G, Su J, Cai H (2024). ^211^At-labeled nanoscale polydopamine decorated with FAPI for synergistic targeted-alpha therapy and photothermal therapy of glioma. ACS Appl Nano Mater.

[62] Liu W, Ma H, Liang R, Chen X, Li H, Lan T (2022). Targeted alpha therapy of glioma using ^211^At-labeled heterodimeric peptide targeting both VEGFR and integrins. Mol Pharm.

[63] Roncali L, Marionneau-Lambot S, Roy C, Eychenne R, Gouard S, Avril S (2024). Brain intratumoural astatine-211 radiotherapy targeting syndecan-1 leads to durable glioblastoma remission and immune memory in female mice. EBioMedicine.

[64] Kato H, Huang X, Kadonaga Y, Katayama D, Ooe K, Shimoyama A (2021). Intratumoral administration of astatine-211-labeled gold nanoparticle for alpha therapy. J Nanobiotechnology.

[65] Liu Y, Zhou Z, Feng Y, Zhao XG, Vaidyanathan G, Zalutsky MR (2021). Gold nanostars: a novel platform for developing ^211^At-labeled agents for targeted alpha-particle therapy. Int J Nanomedicine.

[66] Andersson H, Cederkrantz E, Bäck T, Divgi C, Elgqvist J, Himmelman J (2009). Intraperitoneal α-particle radioimmunotherapy of ovarian cancer patients: pharmacokinetics and dosimetry of ^211^At-MX35 F(ab′)2-a phase I study. J Nucl Med.

[67] Cederkrantz E, Andersson H, Bernhardt P, Bäck T, Hultborn R, Jacobsson L (2015). Absorbed doses and risk estimates of ^211^At-MX35 F(ab')2 in intraperitoneal therapy of ovarian cancer patients. Int J Radiat Oncol Biol Phys.

[68] Hallqvist A, Bergmark K, Bäck T, Andersson H, Dahm-Kähler P, Johansson M (2019). Intraperitoneal α-emitting radioimmunotherapy with ^211^At in relapsed ovarian cancer: long-term follow-up with individual absorbed dose estimations. J Nucl Med.

[69] De Kruijff R, Wolterbeek H, Denkova A (2015). A critical review of alpha radionuclide therapy-how to deal with recoiling daughters?. Pharmaceuticals.

[70] Jaggi JS, Seshan S V, McDevitt MR, LaPerle K, Sgouros G, Scheinberg DA (2005). Renal tubulointerstitial changes after internal irradiation with alpha-particle-emitting actinium daughters. J Am Soc Nephrol.

[71] Miederer M, Scheinberg DA, McDevitt MR (2008). Realizing the potential of the actinium-225 radionuclide generator in targeted alpha particle therapy applications. Adv Drug Deliv Rev.

[72] Deal KA, Davis IA, Mirzadeh S, Kennel SJ, Brechbiel MW (1999). Improved *in vivo* stability of actinium-225 macrocyclic complexes. J Med Chem.

[73] Kratochwil C, Bruchertseifer F, Giesel FL, Weis M, Verburg FA, Mottaghy F (2016). ^225^Ac-PSMA-617 for PSMA-targeted α-radiation therapy of metastatic castration-resistant prostate cancer. J Nucl Med.

[74] Usmani S, Rasheed R, Al Kandari F, Marafi F, Naqvi SAR (2019). ^225^Ac prostate-specific membrane antigen posttherapy imaging: comparing 2 and 3 photopeaks. Clin Nucl Med.

[75] Vatsa R, Sood A, Vadi SK, Das CK, Kaur K, Parmar M (2020). ^225^Ac-PSMA-617 radioligand posttherapy imaging in metastatic castrate-resistant prostate cancer patient using 3 photopeaks. Clin Nucl Med.

[76] Tulik M, Kuliński R, Tabor Z, Brzozowska B, Łaba P, Bruchertseifer F (2024). Quantitative SPECT/CT imaging of actinium-225 for targeted alpha therapy of glioblastomas. EJNMMI Phys.

[77] Melville G, J Allen B (2009). Cyclotron and linac production of Ac-225. Appl Radiat Isot.

[78] Nagatsu K, Suzuki H, Fukada M, Ito T, Ichinose J, Honda Y (2021). Cyclotron production of ^225^Ac from an electroplated ^226^Ra target. Eur J Nucl Med Mol Imaging.

[79] Zimmermann R (2023). Is actinium really happening?. J Nucl Med.

[80] McDevitt MR, Ma D, Lai LT, Simon J, Borchardt P, Frank RK (2001). Tumor therapy with targeted atomic nanogenerators. Science.

[81] McDevitt MR, Ma D, Simon J, Frank RK, Scheinberg DA (2002). Design and synthesis of ^225^Ac radioimmunopharmaceuticals. Appl Radiat and Isot.

[82] Hennrich U, Benešová M (2020). [^68^Ga]Ga-DOTA-TOC: the first FDA-approved ^68^Ga-radiopharmaceutical for PET imaging. Pharmaceuticals.

[83] Chin A, Jiao R, Allen KJH, Li J, Chen M, Vusirikala M (2023). Lintuzumab-Ac225, a CD33-directed antibody radiotherapy, targets AML in a mutation agnostic manner. Blood.

[84] Bidkar AP, Zerefa L, Yadav S, VanBrocklin HF, Flavell RR (2024). Actinium-225 targeted alpha particle therapy for prostate cancer. Theranostics.

[85] Ingham A, Wharton L, Koniar H, Merkens H, McNeil S, Sekar S (2024). Preclinical evaluation of [^225^Ac]Ac-crown-TATE - an alpha-emitting radiopharmaceutical for neuroendocrine tumors. Nucl Med Biol.

[86] Brooks PC, Clark RAF, Cheresh DA (1994). Requirement of vascular integrin alpha v beta 3 for angiogenesis. Science.

[87] Gladson CL (1996). Expression of integrin alpha v beta 3 in small blood vessels of glioblastoma tumors. J Neuropathol Exp Neurol.

[88] Schittenhelm J, Schwab EI, Sperveslage J, Tatagiba M, Meyermann R, Fend F (2013). Longitudinal expression analysis of α_V_ integrins in human gliomas reveals upregulation of integrin α_V_β_3_ as a negative prognostic factor. J Neuropathol Exp Neurol.

[89] Pandya DN, Hantgan R, Budzevich MM, Kock ND, Morse DL, Batista I (2016). Preliminary therapy evaluation of ^225^Ac-DOTA-c(RGDyK) demonstrates that Cerenkov radiation derived from ^225^Ac daughter decay can be detected by optical imaging for *in vivo* tumor visualization. Theranostics.

[90] Shaffer TM, Pratt EC, Grimm J (2017). Utilizing the power of Cerenkov light with nanotechnology. Nat Nanotechnol.

[91] Sattiraju A, Xiong X, Pandya DN, Wadas TJ, Xuan A, Sun Y (2017). Alpha particle enhanced blood brain/tumor barrier permeabilization in glioblastomas using integrin alpha-v beta-3 targeted liposomes. Mol Cancer Ther.

[92] Wang R, Chadalavada K, Wilshire J, Kowalik U, Hovinga KE, Geber A (2010). Glioblastoma stem-like cells give rise to tumour endothelium. Nature.

[93] Maddison K, Bowden NA, Graves MC, Tooney PA (2023). Characteristics of vasculogenic mimicry and tumour to endothelial transdifferentiation in human glioblastoma: a systematic review. BMC Cancer.

[94] Behling K, Maguire WF, Di Gialleonardo V, Heeb LE, Hassan IF, Veach DR (2016). Remodeling the vascular microenvironment of glioblastoma with α-particles. J Nucl Med.

[95] Behling K, Maguire WF, López Puebla JC, Sprinkle SR, Ruggiero A, O'Donoghue J (2016). Vascular targeted radioimmunotherapy for the treatment of glioblastoma. J Nucl Med.

[96] Thaci B, Brown CE, Binello E, Werbaneth K, Sampath P, Sengupta S (2014). Significance of interleukin-13 receptor alpha 2-targeted glioblastoma therapy. Neuro Oncol.

[97] Sattiraju A, Solingapuram Sai KK, Xuan A, Pandya DN, Almaguel FG, Wadas TJ (2017). IL13R_α2_ targeted alpha particle therapy against glioblastomas. Oncotarget.

[98] Yook S, Cai Z, Lu Y, Winnik MA, Pignol JP, Reilly RM (2016). Intratumorally injected ^177^Lu-labeled gold nanoparticles: gold nanoseed brachytherapy with application for neoadjuvant treatment of locally advanced breast cancer. J Nucl Med.

[99] Salvanou EA, Stellas D, Tsoukalas C, Mavroidi B, Paravatou-Petsotas M, Kalogeropoulos N (2020). A proof-of-concept study on the therapeutic potential of au nanoparticles radiolabeled with the alpha-emitter actinium-225. Pharmaceutics.

[100] Wichmann CW, Morgan KA, Cao Z, Osellame LD, Guo N, Gan H (2024). Radiolabeling and preclinical evaluation of therapeutic efficacy of ^225^Ac-ch806 in glioblastoma and colorectal cancer xenograft models. J Nucl Med.

[101] Królicki L, Bruchertseifer F, Kunikowska J, Koziara H, Pawlak D, Kuliński R (2021). Dose escalation study of targeted alpha therapy with [^225^Ac]Ac-DOTA-substance P in recurrence glioblastoma - safety and efficacy. Eur J Nucl Med Mol Imaging.

[102] Hennig IM, Laissue JA, Horisberger U, Reubi JC (1995). Substance-P receptors in human primary neoplasms: tumoral and vascular localization. Int J Cancer.

[103] Palma C, Nardelli F, Manzini S, Maggi CA (1999). Substance P activates responses correlated with tumour growth in human glioma cell lines bearing tachykinin NK1 receptors. Br J Cancer.

[104] Muñoz M, Rosso M, Pérez A, Coveñas R, Rosso R, Zamarriego C (2005). The NK1 receptor is involved in the antitumoural action of L-733,060 and in the mitogenic action of substance P on neuroblastoma and glioma cell lines. Neuropeptides.

[105] Kratochwil C, Apostolidis L, Rathke H, Apostolidis C, Bicu F, Bruchertseifer F (2021). Dosing ^225^Ac-DOTATOC in patients with somatostatin-receptor-positive solid tumors: 5-year follow-up of hematological and renal toxicity. Eur J Nucl Med Mol Imaging.

[106] Jaggi JS, Kappel BJ, McDevitt MR, Sgouros G, Flombaum CD, Cabassa C (2005). Efforts to control the errant products of a targeted *in vivo* generator. Cancer Res.

[107] Schwartz J, Jaggi JS, O'Donoghue JA, Ruan S, McDevitt M, Larson SM (2011). Renal uptake of bismuth-213 and its contribution to kidney radiation dose following administration of actinium-225-labeled antibody. Phys Med Biol.

[108] Morgenstern A, Bruchertseifer F, Apostolidis C (2012). Bismuth-213 and actinium-225 - generator performance and evolving therapeutic applications of two generator-derived alpha-emitting radioisotopes. Curr Radiopharm.

[109] McDevitt MR, Finn RD, Sgouros G, Ma D, Scheinberg DA (1999). An ^225^Ac/^213^Bi generator system for therapeutic clinical applications: construction and operation. Appl Radiat Isot.

[110] Feuerecker B, Michalik M, Hundshammer C, Schwaiger M, Bruchertseifer F, Morgenstern A (2019). Assessment of ^213^Bi-anti-EGFR mAb treatment efficacy in malignant cancer cells with [1-^13^C]pyruvate and [^18^F]FDG. Sci Rep.

[111] Feuerecker B, Biechl P, Seidl C, Bruchertseifer F, Morgenstern A, Schwaiger M (2021). Diverse metabolic response of cancer cells treated with a ^213^Bi-anti-EGFR-immunoconjugate. Sci Rep.

[112] Allen KJH, Jiao R, Malo ME, Frank C, Fisher DR, Rickles D (2019). Comparative radioimmunotherapy of experimental melanoma with novel humanized antibody to melanin labeled with ^213^bismuth and ^177^lutetium. Pharmaceutics.

[113] Chérel M, Gouard S, Gaschet J, Saï-Maurel C, Bruchertseifer F, Morgenstern A (2013). ^213^Bi radioimmunotherapy with an anti-mCD138 monoclonal antibody in a murine model of multiple myeloma. J Nucl Med.

[114] Gustafsson-Lutz A, Bäck T, Aneheim E, Hultborn R, Palm S, Jacobsson L (2017). Therapeutic efficacy of α-radioimmunotherapy with different activity levels of the ^213^Bi-labeled monoclonal antibody MX35 in an ovarian cancer model. EJNMMI Res.

[115] Revskaya E, Jiang Z, Morgenstern A, Bruchertseifer F, Sesay M, Walker S (2017). A radiolabeled fully human antibody to human aspartyl (asparaginyl) β-hydroxylase is a promising agent for imaging and therapy of metastatic breast cancer. Cancer Biother Radiopharm.

[116] Pfost B, Seidl C, Autenrieth M, Saur D, Bruchertseifer F, Morgenstern A (2009). Intravesical α-radioimmunotherapy with ^213^Bi-anti-EGFR-mAb defeats human bladder carcinoma in xenografted nude mice. J Nucl Med.

[117] Autenrieth ME, Seidl C, Bruchertseifer F, Horn T, Kurtz F, Feuerecker B (2018). Treatment of carcinoma *in situ* of the urinary bladder with an alpha-emitter immunoconjugate targeting the epidermal growth factor receptor: a pilot study. Eur J Nucl Med Mol Imaging.

[118] Kneifel S, Cordier D, Good S, Ionescu MC, Ghaffari A, Hofer S (2006). Local targeting of malignant gliomas by the diffusible peptidic vector 1,4,7,10-tetraazacyclododecane-1-glutaric acid-4,7,10-triacetic acid-substance P. Clin Cancer Res.

[119] Cordier D, Forrer F, Bruchertseifer F, Morgenstern A, Apostolidis C, Good S (2010). Targeted alpha-radionuclide therapy of functionally critically located gliomas with ^213^Bi-DOTA-[Thi^8^,Met(O_2_)^11^]-substance P: a pilot trial. Eur J Nucl Med Mol Imaging.

[120] Krolicki L, Bruchertseifer F, Kunikowska J, Koziara H, Królicki B, Jakuciński M (2018). Prolonged survival in secondary glioblastoma following local injection of targeted alpha therapy with ^213^Bi-substance P analogue. Eur J Nucl Med Mol Imaging.

[121] Królicki L, Bruchertseifer F, Kunikowska J, Koziara H, Królicki B, Jakuciński M (2019). Safety and efficacy of targeted alpha therapy with ^213^Bi-DOTA-substance P in recurrent glioblastoma. Eur J Nucl Med Mol Imaging.

[122] Piotrowska A, Męczyńska-Wielgosz S, Majkowska-Pilip A, Koźmiński P, Wójciuk G, Cędrowska E (2017). Nanozeolite bioconjugates labeled with ^223^Ra for targeted alpha therapy. Nucl Med Biol.

[123] Nishri Y, Vatarescu M, Luz I, Epstein L, Dumančić M, Del Mare S (2022). Diffusing alpha-emitters radiation therapy in combination with temozolomide or bevacizumab in human glioblastoma multiforme xenografts. Front Oncol.

[124] Stallons TAR, Saidi A, Tworowska I, Delpassand ES, Torgue JJ (2019). Preclinical investigation of ^212^Pb-DOTAMTATE for peptide receptor radionuclide therapy in a neuroendocrine tumor model. Mol Cancer Ther.

[125] Lizak C, Malvezzi F, Saidi A, Mettier M, Vojackova J, Schibli R (2024). Lead-212 Radio-DARPin Therapeutic (RDT) targeting delta-like ligand 3 (DLL3) shows promising preclinical antitumor efficacy and tolerability in small cell lung cancer (SCLC). J Nucl Med.

[126] Lindland K, Malenge MM, Li RG, Wouters R, Bønsdorff TB, Juzeniene A (2024). Antigen targeting and anti-tumor activity of a novel anti-CD146 ^212^Pb internalizing alpha-radioimmunoconjugate against malignant peritoneal mesothelioma. Sci Rep.

[127] Delpassand ES, Tworowska I, Esfandiari R, Torgue J, Hurt J, Shafie A (2022). Targeted α-emitter therapy with ^212^Pb-DOTAMTATE for the treatment of metastatic SSTR-expressing neuroendocrine tumors: first-in-humans dose-escalation clinical trial. J Nucl Med.

[128] Mawrin C, Schulz S, Pauli SU, Treuheit T, Diete S, Dietzmann K (2004). Differential expression of SST1, SST2A, and SST3 somatostatin receptor proteins in low-grade and high-grade astrocytomas. J Neuropathol Exp Neurol.

[129] Lapa C, Linsenmann T, Lückerath K, Samnick S, Herrmann K, Stoffer C (2015). Tumor-associated macrophages in glioblastoma multiforme-a suitable target for somatostatin receptor-based imaging and therapy?. PLoS One.

[130] Nemati R, Shooli H, Rekabpour SJ, Ahmadzadehfar H, Jafari E, Ravanbod MR (2021). Feasibility and therapeutic potential of peptide receptor radionuclide therapy for high-grade gliomas. Clin Nucl Med.

[131] Karlsson J, Schatz CA, Wengner AM, Hammer S, Scholz A, Cuthbertson A (2023). Targeted thorium-227 conjugates as treatment options in oncology. Front Med (Lausanne).

[132] Favaretto C, Grundler PV, Talip Z, Köster U, Johnston K, Busslinger SD (2024). Terbium-149 production: a focus on yield and quality improvement towards preclinical application. Sci Rep.

[133] Lenting K, Verhaak R, Ter Laan M, Wesseling P, Leenders W (2017). Glioma: experimental models and reality. Acta Neuropathol.

[134] Ledur PF, Onzi GR, Zong H, Lenz G (2017). Culture conditions defining glioblastoma cells behavior: what is the impact for novel discoveries?. Oncotarget.

[135] Evans SM, Judy KD, Dunphy I (2004). Hypoxia is important in the biology and aggression of human glial brain tumors. Clin Cancer Res.

[136] Quan Y, Yan Y, Wang X (2012). Impact of cell dissociation on identification of breast cancer stem cells. Cancer Biomark.

[137] Chen S, So EC, Strome SE, Zhang X (2015). Impact of Detachment Methods on M2 Macrophage Phenotype and Function. J Immunol Methods.

[138] Jager LD, Canda CMA, Hall CA (2016). Effect of enzymatic and mechanical methods of dissociation on neural progenitor cells derived from induced pluripotent stem cells. Adv Med Sci.

[139] Haddad AF, Young JS, Amara D (2021). Mouse models of glioblastoma for the evaluation of novel therapeutic strategies. Neurooncol Adv.

[140] Patil V, Pal J, Somasundaram K (2015). Elucidating the cancer-specific genetic alteration spectrum of glioblastoma derived cell lines from whole exome and RNA sequencing. Oncotarget.

[141] Radaelli E, Ceruti R, Patton V, Russo M, Degrassi A, Croci V (2009). Immunohistopathological and neuroimaging characterization of murine orthotopic xenograft models of glioblastoma multiforme recapitulating the most salient features of human disease. Histol Histopathol.

[142] Chu SH, Zhou ZM, Karri S, Li ZQ, Zhao JM (2014). *In vitro* and *in vivo* radiosensitization of human glioma U251 cells induced by upregulated expression of SLC22A18. Cancer Gene Ther.

[143] Bao S, Wu Q, McLendon RE, Hao Y, Shi Q, Hjelmeland AB (2006). Glioma stem cells promote radioresistance by preferential activation of the DNA damage response. Nature.

[144] Teng J, da Hora CC, Kantar RS, Nakano I, Wakimoto H, Batchelor TT (2017). Dissecting inherent intratumor heterogeneity in patient-derived glioblastoma culture models. Neuro Oncol.

[145] Lee J, Kotliarova S, Kotliarov Y, Li A, Su Q, Donin NM (2006). Tumor stem cells derived from glioblastomas cultured in bFGF and EGF more closely mirror the phenotype and genotype of primary tumors than do serum-cultured cell lines. Cancer Cell.

[146] Patrizii M, Bartucci M, Pine SR, Sabaawy HE (2018). Utility of glioblastoma patient-derived orthotopic xenografts in drug discovery and personalized therapy. Front Oncol.

[147] Alcaniz J, Winkler L, Dahlmann M, Becker M, Orthmann A, Haybaeck J (2023). Clinically relevant glioblastoma patient-derived xenograft models to guide drug development and identify molecular signatures. Front Oncol.

[148] Chupp DP, Rivera CE, Zhou Y, Xu Y, Ramsey PS, Xu Z (2024). A humanized mouse that mounts mature class-switched, hypermutated and neutralizing antibody responses. Nat Immunol.

[149] Wu A, Oh S, Wiesner SM, Ericson K, Chen L, Hall WA (2008). Persistence of CD133^+^ cells in human and mouse glioma cell lines: detailed characterization of GL261 glioma cells with cancer stem cell-like properties. Stem Cells Dev.

[150] Genoud V, Marinari E, Nikolaev SI, Castle JC, Bukur V, Dietrich PY (2018). Responsiveness to anti-PD-1 and anti-CTLA-4 immune checkpoint blockade in SB28 and GL261 mouse glioma models. Oncoimmunology.

[151] Guan X, Hasan MN, Begum G, Kohanbash G, Carney KE, Pigott VM (2018). Blockade of Na/H exchanger stimulates glioma tumor immunogenicity and enhances combinatorial TMZ and anti-PD-1 therapy. Cell Death Dis.

[152] Gonzalez-Junca A, Reiners O, Borrero-Garcia LD, Beckford-Vera D, Lazar AA, Chou W (2021). Positron emission tomography imaging of functional transforming growth factor β (TGFβ) activity and benefit of TGFβ inhibition in irradiated intracranial tumors. Int J Radiat Oncol Biol Phys.

[153] Miyai M, Tomita H, Soeda A, Yano H, Iwama T, Hara A (2017). Current trends in mouse models of glioblastoma. J Neurooncol.

[154] Stribbling SM, Ryan AJ (2022). The cell-line-derived subcutaneous tumor model in preclinical cancer research. Nat Protoc.

[155] Le Reste PJ, Pineau R, Voutetakis K, Samal J, Jégou G, Lhomond S (2020). Local intracerebral inhibition of IRE1 by MKC8866 sensitizes glioblastoma to irradiation/chemotherapy *in vivo*. Cancer Lett.

[156] Vanpouille-Box C, Lacoeuille F, Belloche C, Lepareur N, Lemaire L, LeJeune JJ (2011). Tumor eradication in rat glioma and bypass of immunosuppressive barriers using internal radiation with ^188^Re-lipid nanocapsules. Biomaterials.

[157] Resende FFB, Bai X, Del Bel EA, Kirchhoff F, Scheller A, Titze-de-Almeida R (2016). Evaluation of TgH(CX3CR1-EGFP) mice implanted with mCherry-GL261 cells as an *in vivo* model for morphometrical analysis of glioma-microglia interaction. BMC Cancer.

[158] Irtenkauf SM, Sobiechowski S, Hasselbach LA, Nelson KK, Transou AD, Carlton ET (2017). Optimization of glioblastoma mouse orthotopic xenograft models for translational research. Comp Med.

[159] Assi H, Candolfi M, Lowenstein PR, Castro MG (2012). Rodent glioma models: intracranial stereotactic allografts and xenografts. Neuromethods.

[160] Sarkaria JN, Hu LS, Parney IF, Pafundi DH, Brinkmann DH, Laack NN (2018). Is the blood-brain barrier really disrupted in all glioblastomas? A critical assessment of existing clinical data. Neuro Oncol.

[161] Lonser RR, Sarntinoranont M, Morrison PF, Oldfield EH (2015). Convection-enhanced delivery to the central nervous system. J Neurosurg.

[162] D'Amico RS, Aghi MK, Vogelbaum MA, Bruce JN (2021). Convection-enhanced drug delivery for glioblastoma: a review. J Neurooncol.

[163] Larsen RH, Slade S, Zalutsky MR (1998). Blocking [^211^At]astatide accumulation in normal tissues: preliminary evaluation of seven potential compounds. Nucl Med Biol.

[164] Lundh C, Lindencrona U, Schmitt A, Nilsson M, Forssell-Aronsson E (2006). Biodistribution of free ^211^At and ^125^I- in nude mice bearing tumors derived from anaplastic thyroid carcinoma cell lines. Cancer Biother Radiopharm.

[165] Spetz J, Rudqvist N, Forssell-Aronsson E (2013). Biodistribution and dosimetry of free ^211^At, ^125^I- and ^131^I- in rats. Cancer Biother Radiopharm.

[166] Tronchin S, Forster JC, Hickson K, Bezak E (2022). Dosimetry in targeted alpha therapy. A systematic review: current findings and what is needed. Phys Med Biol.

[167] Amatori S, Tavolaro S, Gambardella S, Fanelli M (2021). The dark side of histones: genomic organization and role of oncohistones in cancer. Clin Epigenetics.

[168] Shapiro WR, Carpenter SP, Roberts K, Shan JS (2006). ^131^I-chTNT-1/B mAb: tumour necrosis therapy for malignant astrocytic glioma. Expert Opin Biol Ther.

[169] Hdeib A, Sloan A (2012). Targeted radioimmunotherapy: the role of ^131^I-chTNT-1/B mAb (Cotara) for treatment of high-grade gliomas. Future Oncol.

[170] Richardson PJ (2016). CXCR4 and glioblastoma. Anticancer Agents Med Chem.

[171] Han JH, Yoon JS, Chang DY, Cho KG, Lim J, Kim SS (2020). CXCR4-STAT3 axis plays a role in tumor cell infiltration in an orthotopic mouse glioblastoma model. Mol Cells.

[172] Khan AB, Lee S, Harmanci AS, Patel R, Latha K, Yang Y (2023). CXCR4 expression is associated with proneural-to-mesenchymal transition in glioblastoma. Int J Cancer.

[173] Séhédic D, Chourpa I, Tétaud C, Griveau A, Loussouarn C, Avril S (2017). Locoregional confinement and major clinical benefit of ^188^Re-loaded CXCR4-targeted nanocarriers in an orthotopic human to mouse model of glioblastoma. Theranostics.

[174] Škrlec K, Štrukelj B, Berlec A (2015). Non-immunoglobulin scaffolds: a focus on their targets. Trends Biotechnol.

[175] Cheal SM, Chung SK, Vaughn BA, Cheung NV, Larson SM (2022). Pretargeting: a path forward for radioimmunotherapy. J Nucl Med.

[176] Paganelli G, Grana C, Chinol M, Cremonesi M, De Cicco C, De Braud F (1999). Antibody-guided three-step therapy for high grade glioma with yttrium-90 biotin. Eur J Nucl Med.

[177] Moosmayer D, Berndorff D, Chang CH, Sharkey RM, Rother A, Borkowski S (2006). Bispecific antibody pretargeting of tumor neovasculature for improved systemic radiotherapy of solid tumors. Clin Cancer Res.

[178] Chung SK, Vargas DB, Chandler CS, Katugampola S, Veach DR, McDevitt MR (2023). Efficacy of HER2-targeted intraperitoneal ^225^Ac α-pretargeted radioimmunotherapy for small-volume ovarian peritoneal carcinomatosis. J Nucl Med.

[179] Choi JW, Seo M, Kim K, Kim AR, Lee H, Kim HS (2023). Aptamer nanoconstructs crossing human blood-brain barrier discovered *via* microphysiological system-based SELEX technology. ACS Nano.

[180] Hicke BJ, Stephens AW, Gould T, Chang YF, Lynott CK, Heil J (2006). Tumor targeting by an aptamer. J Nucl Med.

[181] Doherty C, Wilbanks B, Khatua S, Maher LJ (2024). Aptamers in neuro-oncology: an emerging therapeutic modality. Neuro Oncol.

[182] Laber DA, Choudry MA, Taft BS, Bhupalam L, Sharma VR, Hendler FJ (2004). A phase I study of AGRO100 in advanced cancer. J Clin Oncol.

[183] Laber DA, Sharma VR, Bhupalam L, Taft B, Hendler FJ, Barnhart KM (2005). Update on the first phase I study of AGRO100 in advanced cancer. J Clin Oncol.

[184] Rosenberg JE, Bambury RM, Van Allen EM, Drabkin HA, Lara PN Jr, Harzstark AL (2014). A phase II trial of AS1411 (a novel nucleolin-targeted DNA aptamer) in metastatic renal cell carcinoma. Invest New Drugs.

[185] Zhang X, Peng L, Liang Z, Kou Z, Chen Y, Shi G (2018). Effects of aptamer to U87-EGFRvIII cells on the proliferation, radiosensitivity, and radiotherapy of glioblastoma cells. Mol Ther Nucleic Acids.

[186] Wang L, Wang N, Zhang W, Cheng X, Yan Z, Shao G (2022). Therapeutic peptides: current applications and future directions. Signal Transduct Targeted Ther.

[187] Kiviniemi A, Gardberg M, Frantzén J, Pesola M, Vuorinen V, Parkkola R (2015). Somatostatin receptor subtype 2 in high-grade gliomas: PET/CT with ^68^Ga-DOTA-peptides, correlation to prognostic markers, and implications for targeted radiotherapy. EJNMMI Res.

[188] Singh S, Halperin D, Myrehaug S, Herrmann K, Pavel M, Kunz PL (2024). [^177^Lu]Lu-DOTA-TATE plus long-acting octreotide versus high-dose long-acting octreotide for the treatment of newly diagnosed, advanced grade 2-3, well-differentiated, gastroenteropancreatic neuroendocrine tumours (NETTER-2): an open-label, randomised, phase 3 study. Lancet.

[189] Hänscheid H, Schirbel A, Hartrampf P, Kraus S, Werner RA, Einsele H (2022). Biokinetics and dosimetry of ^177^Lu-pentixather. J Nucl Med.

[190] Chen Z, Xue Q, Yao S (2023). Nuclear medicine application of pentixafor/pentixather targeting CXCR4 for imaging and therapy in related disease. Mini Rev Med Chem.

[191] Moody TW, Lee L, Ramos-Alvarez I, Iordanskaia T, Mantey SA, Jensen RT (2021). Bombesin receptor family activation and CNS/neural tumors: review of evidence supporting possible role for novel targeted therapy. Front Endocrinol (Lausanne).

[192] Galbo PM Jr, Madsen AT, Liu Y, Peng M, Wei Y, Ciesielski MJ (2024). Functional contribution and clinical implication of cancer-associated fibroblasts in glioblastoma. Clinical cancer research.

[193] Jeanjean P, Kwock S, Tse C, Cloughesy T, Czernin J, Carlucci G (2022). Fibroblast activation protein (FAP) as a target for radioligand therapy in glioblastoma. J Nucl Med.

[194] Bhamidipati D, Haro-Silerio JI, Yap TA, Ngoi N (2023). PARP inhibitors: enhancing efficacy through rational combinations. Br J Cancer.

[195] Jannetti SA, Carlucci G, Carney B, Kossatz S, Shenker L, Carter LM (2018). PARP-1-targeted radiotherapy in mouse models of glioblastoma. J Nucl Med.

[196] Wang JH, Kiess AP (2023). PSMA-targeted therapy for non-prostate cancers. Front Oncol.

[197] Kunikowska J, Charzyńska I, Kuliński R, Pawlak D, Maurin M, Królicki L (2020). Tumor uptake in glioblastoma multiforme after IV injection of [^177^Lu]Lu-PSMA-617. Eur J Nucl Med Mol Imaging.

[198] Kumar A, Ballal S, Yadav MP, ArunRaj ST, Haresh KP, Gupta S (2020). ^177^Lu-/^68^Ga-PSMA theranostics in recurrent glioblastoma multiforme: proof of concept. Clin Nucl Med.

[199] Kiess AP, Minn I, Vaidyanathan G, Hobbs RF, Josefsson A, Shen C (2016). (2S)-2-(3-(1-Carboxy-5-(4-^211^At-Astatobenzamido)Pentyl)Ureido)-pentanedioic acid for PSMA-targeted α-particle radiopharmaceutical therapy. J Nucl Med.

[200] Mease RC, Kang CM, Kumar V, Banerjee SR, Minn I, Brummet M (2022). An improved ^211^At-labeled agent for PSMA-targeted α-therapy. J Nucl Med.

[201] Feng Y, Meshaw RL, Finch SW, Zheng Y, Minn I, Vaidyanathan G (2024). A third generation PSMA-targeted agent [^211^At]YF2: synthesis and *in vivo* evaluation. Nucl Med Biol.

[202] Shultz MD, Wilson JD, Fuller CE, Zhang J, Dorn HC, Fatouros PP (2011). Metallofullerene-based nanoplatform for brain tumor brachytherapy and longitudinal imaging in a murine orthotopic xenograft model. Radiology.

[203] Phillips WT, Goins B, Bao A, Vargas D, Guttierez JE, Trevino A (2012). Rhenium-186 liposomes as convection-enhanced nanoparticle brachytherapy for treatment of glioblastoma. Neuro Oncol.

[204] Salvanou EA, Kolokithas-Ntoukas A, Liolios C, Xanthopoulos S, Paravatou-Petsotas M, Tsoukalas C (2022). Preliminary evaluation of iron oxide nanoparticles radiolabeled with ^68^Ga and ^177^Lu as potential theranostic agents. Nanomaterials.

[205] Salvanou EA, Kolokithas-Ntoukas A, Prokopiou D, Theodosiou M, Efthimiadou E, Koźmiński P (2024). ^177^lu-labeled iron oxide nanoparticles functionalized with doxorubicin and bevacizumab as nanobrachytherapy agents against breast cancer. Molecules.

[206] Bandekar A, Zhu C, Jindal R, Bruchertseifer F, Morgenstern A, Sofou S (2014). Anti-prostate-specific membrane antigen liposomes loaded with ^225^Ac for potential targeted antivascular α-particle therapy of cancer. J Nucl Med.

[207] Toro-González M, Akingbesote N, Bible A, Pal D, Sanders B, Ivanov AS (2024). Development of ^225^Ac-doped biocompatible nanoparticles for targeted alpha therapy. J Nanobiotechnology.

[208] Chen X, Liang R, Liu W, Ma H, Bai C, Xiong Y (2023). Biocompatible conjugated polymer nanoparticles labeled with ^225^Ac for tumor endoradiotherapy. Bioorg Med Chem.

[209] Wang R, Wolterbeek HTh, Denkova AG (2024). Lead-212/bismuth-212 *in vivo* generator based on ultrasmall silver telluride nanoparticles. J Labelled Comp Radiopharm.

[210] Gregory JV, Kadiyala P, Doherty R, Cadena M, Habeel S, Ruoslahti E (2020). Systemic brain tumor delivery of synthetic protein nanoparticles for glioblastoma therapy. Nat Commun.

[211] Obata H, Ogawa M, Zalutsky MR (2023). DNA repair inhibitors: potential targets and partners for targeted radionuclide therapy. Pharmaceutics.

[212] Gorin JB, Ménager J, Gouard S, Maurel C, Guilloux Y, Faivre-Chauvet A (2014). Antitumor immunity induced after α irradiation. Neoplasia.

[213] Malamas AS, Gameiro SR, Knudson KM, Hodge JW (2016). Sublethal exposure to alpha radiation (^223^Ra dichloride) enhances various carcinomas' sensitivity to lysis by antigenspecific cytotoxic T lymphocytes through calreticulin-mediated immunogenic modulation. Oncotarget.

[214] Lejeune P, Cruciani V, Berg-Larsen A, Schlicker A, Mobergslien A, Bartnitzky L (2021). Immunostimulatory effects of targeted thorium-227 conjugates as single agent and in combination with anti-PD-L1 therapy. J Immunother Cancer.

[215] Perrin J, Capitao M, Allard M, Chouin N, Gouard S, Marionneau-Lambot S (2022). Targeted alpha particle therapy remodels the tumor microenvironment and improves efficacy of immunotherapy. Int J Radiat Oncol Biol Phys.

[216] Urbanska AM, Khanin R, Alidori S, Wong S, Mello BP, Almeida BA (2020). A genomic profile of local immunity in the melanoma microenvironment following treatment with α particle-emitting ultrasmall silica nanoparticles. Cancer Biother Radiopharm.

[217] Kim JW, Shin MS, Kang Y, Kang I, Petrylak DP (2018). Immune analysis of radium-223 in patients with metastatic prostate cancer. Clin Genitourin Cancer.

[218] Bellia SR, Feliciani G, Duca MD, Monti M, Turri V, Sarnelli A (2019). Clinical evidence of abscopal effect in cutaneous squamous cell carcinoma treated with diffusing alpha emitters radiation therapy: a case report. J Contemp Brachytherapy.

[219] Li M, Liu D, Lee D, Cheng Y, Baumhover NJ, Marks BM (2021). Targeted alpha-particle radiotherapy and immune checkpoint inhibitors induces cooperative inhibition on tumor growth of malignant melanoma. Cancers (Basel).

[220] Vardaki I, Corn P, Gentile E, Song JH, Madan N, Hoang A (2021). Radium-223 treatment increases immune checkpoint expression in extracellular vesicles from the metastatic prostate cancer bone microenvironment. Clin Cancer Res.

[221] Malo ME, Allen KJH, Jiao R, Frank C, Rickles D, Dadachova E (2020). Mechanistic insights into synergy between melanin-targeting radioimmunotherapy and immunotherapy in experimental melanoma. Int J Mol Sci.

[222] Jiao R, Allen KJH, Malo ME, Rickles D, Dadachova E (2020). Evaluating the combination of radioimmunotherapy and immunotherapy in a melanoma mouse model. Int J Mol Sci.

[223] Liau LM, Ashkan K, Brem S, Campian JL, Trusheim JE, Iwamoto FM (2023). Association of autologous tumor lysate-loaded dendritic cell vaccination with extension of survival among patients with newly diagnosed and recurrent glioblastoma: a phase 3 prospective externally controlled cohort trial. JAMA Oncol.

[224] Bagley SJ, Logun M, Fraietta JA, Wang X, Desai AS, Bagley LJ (2024). Intrathecal bivalent CAR T cells targeting EGFR and IL13R_α2_ in recurrent glioblastoma: phase 1 trial interim results. Nat Med.

[225] Liu Y, Zhou F, Ali H, Lathia JD, Chen P (2024). Immunotherapy for glioblastoma: current state, challenges, and future perspectives. Cell Mol Immunol.

[226] Bellavia MC, Patel RB, Anderson CJ (2022). Combined targeted radiopharmaceutical therapy and immune checkpoint blockade: from preclinical advances to the clinic. J Nucl Med.

[227] Matsuo M, Miwa K, Tanaka O, Shinoda J, Nishibori H, Tsuge Y (2012). Impact of [11C]methionine positron emission tomography for target definition of glioblastoma multiforme in radiation therapy planning. Int J Radiat Oncol Biol Phys.

[228] Mo F, Pellerino A, Soffietti R, Rudà R (2021). Blood-brain barrier in brain tumors: biology and clinical relevance. Int J Mol Sci.

[229] Wen L, Tan Y, Dai S, Zhu Y, Meng T, Yang X (2017). VEGF-mediated tight junctions pathological fenestration enhances doxorubicin-loaded glycolipid-like nanoparticles traversing BBB for glioblastoma-targeting therapy. Drug Deliv.

[230] Zhao C, Wang H, Xiong C, Liu Y (2018). Hypoxic glioblastoma release exosomal VEGF-A induce the permeability of blood-brain barrier. Biochem Biophys Res Commun.

[231] Wen PY, Macdonald DR, Reardon DA, Cloughesy TF, Sorensen AG, Galanis E (2010). Updated response assessment criteria for high-grade gliomas: response assessment in neuro-oncology working group. J Clin Oncol.

[232] Santarosa C, Castellano A, Conte GM, Cadioli M, Iadanza A, Terreni MR (2016). Dynamic contrast-enhanced and dynamic susceptibility contrast perfusion MR imaging for glioma grading: preliminary comparison of vessel compartment and permeability parameters using hotspot and histogram analysis. Eur J Radiol.

[233] Ware JB, Sinha S, Morrison J, Walter AE, Gugger JJ, Schneider ALC (2022). Dynamic contrast enhanced MRI for characterization of blood-brain-barrier dysfunction after traumatic brain injury. Neuroimage Clin.

[234] Yang Y, He MZ, Li T, Yang X (2017). MRI combined with PET-CT of different tracers to improve the accuracy of glioma diagnosis: a systematic review and meta-analysis. Neurosurg Rev.

[235] Arvanitis CD, Ferraro GB, Jain RK (2020). The blood-brain barrier and blood-tumour barrier in brain tumours and metastases. Nat Rev Cancer.

[236] Shan H, Zheng G, Bao S, Yang H, Shrestha UD, Li G (2024). Tumor perfusion enhancement by focus ultrasound-induced blood-brain barrier opening to potentiate anti-PD-1 immunotherapy of glioma. Transl Oncol.

[237] Sabbagh A, Beccaria K, Ling X, Marisetty A, Ott M, Caruso H (2021). Opening of the blood-brain barrier using low-intensity pulsed ultrasound enhances responses to immunotherapy in preclinical glioma models. Clin Cancer Res.

[238] Tao W, Farokhzad OC (2022). Theranostic nanomedicine in the NIR-II window: classification, fabrication, and biomedical applications. Chem Rev.

[239] Cai Q, Li X, Xiong H, Fan H, Gao X, Vemireddy V (2023). Optical blood-brain-tumor barrier modulation expands therapeutic options for glioblastoma treatment. Nat Commun.

[240] Sonabend AM, Gould A, Amidei C, Ward R, Schmidt KA, Zhang DY (2023). Repeated blood-brain barrier opening with an implantable ultrasound device for delivery of albumin-bound paclitaxel in patients with recurrent glioblastoma: a phase 1 trial. Lancet Oncol.

[241] Carpentier A, Stupp R, Sonabend AM, Dufour H, Chinot O, Mathon B (2024). Repeated blood-brain barrier opening with a nine-emitter implantable ultrasound device in combination with carboplatin in recurrent glioblastoma: a phase I/II clinical trial. Nat Commun.

[242] Sharma D, Leong KX, Czarnota GJ (2022). Application of ultrasound combined with microbubbles for cancer therapy. Int J Mol Sci.

[243] Sun L, Joh DY, Al-Zaki A, Stangl M, Murty S, Davis JJ (2016). Theranostic application of mixed gold and superparamagnetic iron oxide nanoparticle micelles in glioblastoma multiforme. J Biomed Nanotechnol.

[244] Guerra DB, Oliveira EMN, Sonntag AR, Sbaraine P, Fay AP, Morrone FB (2022). Intercomparison of radiosensitization induced by gold and iron oxide nanoparticles in human glioblastoma cells irradiated by 6 MV photons. Sci Rep.

[245] Li Q, Qian W, Zhang Y, Hu L, Chen S, Xia Y (2023). A new wave of innovations within the DNA damage response. Signal Transduct Targeted Ther.

[246] Huang RX, Zhou PK (2020). DNA damage response signaling pathways and targets for radiotherapy sensitization in cancer. Signal Transduct Targeted Ther.

[247] Yi G, Sung Y, Kim C, Ra JS, Hirakawa H, Kato TA (2023). DNA polymerase θ-mediated repair of high LET radiation-induced complex DNA double-strand breaks. Nucleic Acids Res.

[248] Everix L, Nair S, Driver CHS, Goethals I, Sathekge MM, Ebenhan T (2022). Perspective on the use of DNA repair inhibitors as a tool for imaging and radionuclide therapy of glioblastoma. Cancers (Basel).

[249] Makvandi M, Lee H, Puentes LN, Reilly SW, Rathi KS, Weng CC (2019). Targeting PARP-1 with alpha-particles is potently cytotoxic to human neuroblastoma in preclinical models. Mol Cancer Ther.

[250] Goyal A, Bauer J, Hey J, Papageorgiou DN, Stepanova E, Daskalakis M (2023). DNMT and HDAC inhibition induces immunogenic neoantigens from human endogenous retroviral element-derived transcripts. Nat Commun.

[251] Huang W, Zhu Q, Shi Z, Tu Y, Li Q, Zheng W (2024). Dual inhibitors of DNMT and HDAC induce viral mimicry to induce antitumour immunity in breast cancer. Cell Death Discov.

[252] Ostuni E, Taylor MRG (2023). Commercial and business aspects of alpha radioligand therapeutics. Front Med (Lausanne).

